# Intrinsic Noise Induces Critical Behavior in Leaky Markovian Networks Leading to Avalanching

**DOI:** 10.1371/journal.pcbi.1003411

**Published:** 2014-01-09

**Authors:** Garrett Jenkinson, John Goutsias

**Affiliations:** Whitaker Biomedical Engineering Institute, The Johns Hopkins University, Baltimore, Maryland, United States of America; Indiana University, United States of America

## Abstract

The role intrinsic statistical fluctuations play in creating avalanches – patterns of complex bursting activity with scale-free properties – is examined in leaky Markovian networks. Using this broad class of models, we develop a probabilistic approach that employs a potential energy landscape perspective coupled with a macroscopic description based on statistical thermodynamics. We identify six important thermodynamic quantities essential for characterizing system behavior as a function of network size: the internal potential energy, entropy, free potential energy, internal pressure, pressure, and bulk modulus. In agreement with classical phase transitions, these quantities evolve smoothly as a function of the network size until a critical value is reached. At that value, a discontinuity in pressure is observed that leads to a spike in the bulk modulus demarcating loss of thermodynamic robustness. We attribute this novel result to a reallocation of the ground states (global minima) of the system's stationary potential energy landscape caused by a noise-induced deformation of its topographic surface. Further analysis demonstrates that appreciable levels of intrinsic noise can cause avalanching, a complex mode of operation that dominates system dynamics at near-critical or subcritical network sizes. Illustrative examples are provided using an epidemiological model of bacterial infection, where avalanching has not been characterized before, and a previously studied model of computational neuroscience, where avalanching was erroneously attributed to specific neural architectures. The general methods developed here can be used to study the emergence of avalanching (and other complex phenomena) in many biological, physical and man-made interaction networks.

## Author Summary

Networks of noisy interacting components arise in diverse scientific disciplines. Here, we develop a mathematical framework to study the underlying causes of a bursting phenomenon in network activity known as avalanching. As prototypical examples, we study a model of disease spreading in a population of individuals and a model of brain activity in a neural network. Although avalanching is well-documented in neural networks, thought to be crucial for learning, information processing, and memory, it has not been studied before in disease spreading. We employ tools originally used to analyze thermodynamic systems to argue that randomness in the actions of individual network components plays a fundamental role in avalanche formation. We show that avalanching is a spontaneous behavior, brought about by a phenomenon reminiscent to a phase transition in statistical mechanics, caused by increasing randomness as the network size decreases. Our work demonstrates that a previously suggested balanced feed-forward network structure is not necessary for neuronal avalanching. Instead, we attribute avalanching to a reallocation of the global minima of the network's stationary potential energy landscape, caused by a noise-induced deformation of its topographic surface.

## Introduction

An important problem in many scientific disciplines is understanding how extrinsic and intrinsic factors enable a complex physical system to exhibit a bursting behavior that leads to avalanching [1], [2]. Avalanching is a form of spontaneous behavior characterized by irregular and isolated bursts of activity that follow a scale-free distribution typical to systems near criticality. In the brain, this mode of operation is thought to play a crucial role in information processing, memory, and learning [2]–[6].

Although avalanche dynamics have been extensively studied *in vitro* [7] and *in vivo* [8], [9] for cortical neural networks, it is not clear what causes avalanching. A recent *in silico* attempt to address this issue [10] was based on approximating the dynamics of a Markovian model of nonlinear interactions between noisy excitatory and inhibitory neurons by Gaussian fluctuations around the macroscopic system behavior using the linear noise approximation (LNA) method of van Kampen [11]. This led to the conclusion that the cause of neural avalanches is a balanced feed-forward (BFF) network structure. We argue here that the Gaussian approximation used to arrive at this conclusion is not appropriate for studying avalanching, thus leading to deficient results. As a consequence, understanding the underlying causes of avalanching *in silico* is still an open problem.

To address this challenge, we introduce a theoretical framework that allows us to examine the role of intrinsic noise in inducing critical behavior that leads to avalanching. Although the idea that noise may induce avalanching has been proposed more that a decade ago [12], our framework leads to a novel understanding of the underlying causes of avalanching in a particular class of complex networks. We focus on a general Markovian network model, which we term leaky Markovian network (LMN), with binary-valued 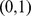 state dynamics. These dynamics are described by a time-dependent probability distribution that evolves according to a well-defined master equation [13] (see Methods for details). It turns out that a LMN is a continuous-time stochastic Boolean network model with a state-dependent asynchronous node updating scheme (we provide details in Text S1). LMNs can model a number of natural and man-made systems of interacting species, such as genetic, neural, epidemiological, and social networks.

Recent work has clearly demonstrated the importance of stochastically modeling physical systems using Markovian networks. The main reason is that intrinsic noise produced by these networks may induce behavior not accounted for by deterministic models [14]–[17]. Examples of such behavior include the emergence of noise-induced modes, stochastic transitions between different operational states, and “stabilization” of existing modes.

In this paper, we study the effect of intrinsic noise on avalanching by using a LMN model. We do so by employing the notion of potential energy landscape [13], [18], [19] and by establishing a connection between statistical thermodynamics and the kinetics of bursting. We quantify the landscape by calculating logarithms of the ratios between the stationary probabilities of individual states and the stationary probability of the most probable state. To reduce computational complexity, we follow a coarse graining approach that transforms the original LMN model into another (non-binary) LMN model with appreciably smaller state-space. To accomplish this task, we partition the nodes of the LMN into homogeneous subpopulations and characterize system behavior by using the dynamic evolution of the *fractional activity process*, which quantifies the fraction of active nodes (nodes with value 1) in each subpopulation. Moreover, we parameterize the LMN in terms of the network size 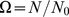, where 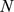 is the net number of nodes in the network and 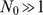 is a normalizing constant such that 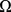 can be approximately considered to be continuous-valued. We refer the reader to the Methods section for details.

The behavior of the fractional activity process is fundamentally affected by 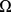. In general, the strength of stochastic fluctuations (intrinsic noise) in the activity process may be thought of as the probability of moving uphill on a fixed potential energy surface, which decays exponentially with increasing 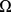. At sufficiently large network sizes 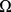, the LMN operates around a ground state of the potential surface located at a fixed point 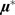 predicted by the macroscopic equations associated with the LNA method, which we assume to be unique, nonzero, and stable (see Methods and Text S1 for details). In this case, a new mode of operation is introduced in the system, as the network size decreases, in the form of a potential well in the topographic surface of the energy landscape, located at the *inactive* state 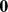. This is a “noise-induced” mode, since it appears at small network sizes at which the fractional activity process is subject to appreciable intrinsic fluctuations.

We show in this paper that noise-induced deformation of the stationary potential energy landscape is the underlying cause of avalanching in LMNs. For sufficiently large network sizes, the potential energy landscape can be approximated by a quadratic surface centered at 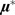. In this case, the LMN operates within the potential well associated with this mode, except for rare and brief random excursions away from that mode. As a consequence, the fractional activity process will fluctuate in a Gaussian-like manner around the macroscopic mode. At smaller network sizes, the fractional activity process is characterized by a bistable behavior between the macroscopic and noise-induced modes, spending most time within the potential well associated with the macroscopic mode, at which the potential energy surface attains its global minimum, while occasionally jumping inside the potential well associated with the noise-induced mode at 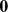. As a consequence, the fractional activity dynamics take on a bursting behavior characterized by long periods of appreciable activity followed by short periods of minimal (almost zero) activity. When the network size decreases further, the noise-induced mode becomes the main stable operating point (i.e., the point at which the potential energy surface attains its global minimum), whereas the macroscopic mode becomes shallower and eventually disappears. In this case, the system is trapped within the potential well associated with the noise-induced mode, except for random and brief excursions away from that mode. As a consequence, the fractional activity process will still exhibit bursting, but now characterized by long periods of minimal (almost zero) activity followed by short bursts of appreciable activity.

Thermodynamic analysis reveals critical behavior in LMNs (we provide details in the Methods section and Text S1). By employing a number of statistical thermodynamic quantities, such as internal and free potential energies, entropy, internal pressure, pressure and bulk modulus (inverse compressibility), we effectively summarize the stochastic behavior of a LMN as its size 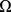 decreases to zero. We also use these summaries to quantify network robustness and the stability of a given state. In agreement with the classical theory of phase transitions, the previous thermodynamic quantities evolve smoothly as a function of 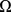 until a critical network size 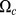 is reached. At this size, a discontinuity is observed in the system pressure, which produces a spike in the bulk modulus demarcating loss of thermodynamic robustness. Critical behavior is caused by reallocation of the ground states (global minima) of the potential energy landscape due to noise-induced deformation of its topographic surface. In particular, observed critical behavior produces two distinct phases: one in which the fixed point 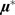 predicted by the macroscopic equations associated with the LNA method constitutes the ground state of the potential energy landscape and one in which the ground state is reallocated to the noise-induced mode at 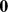. We conclude that avalanching is a complex mode of operation that dominates system dynamics at near-critical and subcritical network sizes due to deformations of the potential energy landscape as the network size decreases to zero, caused by appreciable levels of intrinsic noise.

It is important to mention here that our work provides a novel stochastic perspective to the well-known phenomenon of self-organized criticality (SOC) [20]; i.e., the spontaneous emergence of critical behavior without tuning system parameters whose values are influenced by external factors. We approach SOC from the perspective of nonlinear Markovian dynamics and directly associate self-organization properties of a complex network with the existence of a unique probability distribution at steady-state, which leads to a unique stationary potential energy landscape. Our work demonstrates that SOC may emerge as a consequence of two interweaved adaptive processes that may take place on separate timescales [21], [22]: a short timescale convergence to dynamic equilibrium (stationarity), during which the topological structure of a neural network subsystem is kept relatively fixed (is quasistatic), and longer timescale alterations in topological properties that lead to changes in the size of the network. For example, the neural activities modeled by our LMNs occur on a short timescale compared to the timescale of neural development, where it is well known that programmed cell death plays a major role [23]. This large reduction in the number of neurons could presumably serve to bring neural subsystems within proximity of their critical sizes in accordance with their underlying connectivity structures. On the other hand, an adaptive interplay between the short timescale neural dynamics and the quasistatic topological network structure may lead to a longer timescale topological self-organization that enlarges or contracts the neural subsystem with an objective to keep the system robustly close to criticality [22]. We show that such long timescale alterations can result in a spontaneous reallocation of the ground states in the stationary potential energy landscape due to a noise-induced deformation of its topographic surface. Our approach associates SOC with observable stochastic multistability, which is directly related to the phenomenon of phase transition, thus bridging the gap between “self-organized” and “classical” criticality (see also [24]).

Finally, we would like to point out that, in the neural network literature, it is commonly said that avalanching occurs in the *supercritical* regime near the critical point. On the other hand, we show in this paper that avalanching occurs in a *subcritical* regime near the critical point. To avoid confusion, the reader must keep in mind that these statements do not contradict each other. Correctly using the terms “subcritical” and “supercritical” depends on the parameter employed to take a system from one regime to the other. Contrary to existing works that study criticality in terms of functional parameters (e.g., in terms of the firing rate of all neurons in a neural network), we study in this paper criticality in terms of a structural parameter, the system size, which is inversely related to the strength of intrinsic noise. In this case, avalanching occurs at network sizes smaller but near a critical value, which forces us to use the aforementioned terminology.

## Methods

### Leaky Markovian networks

We consider a directed weighted network 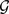 with 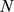 nodes from a set 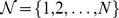, characterized by an 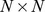 adjacency matrix 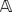. The element 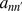 of this matrix assigns a value to the edge leaving the 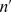-th node and entering the 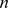-th node whose importance will become clear shortly. Each node represents a species (e.g., an individual or neuron) which, in some well-defined sense, can be active or inactive at time 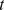 with some probability. We use 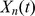 to denote the state of the 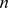-th node of the network at time 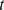, taking value 1 if the node is active and 0 if the node is inactive. Then, we represent the state dynamics of the network by an 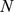-dimensional random process 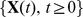 whose 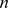-th element 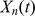 takes binary 0–1 values. We refer to 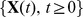 as the activity process.

We assume that, within an infinitesimally small time interval 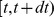, the state of the 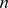-th node is influenced by the net input 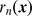 to the node, where 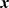 is the state of the network 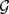 at time 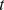 and 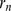 is a real-valued scalar function. In particular, we assume that the probability of the 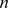-th node to transition from the inactive to the active state within 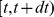 is proportional to 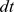, given by 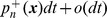, where 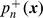 is known as the propensity function and 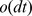 is a term that goes to zero faster than 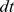. We set
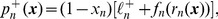
(1)for some nonnegative parameter 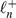 and a nonnegative function 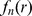. The term 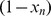 ensures that transition to the active state is possible only when the 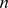-th node is inactive (i.e., when 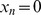), whereas the function 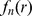 describes how the net input affects the probability of transition. On the other hand, when 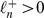, the parameter 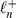 forces the node to be “leaky,” in the sense that it has a fixed propensity to transition from the inactive to the active state, even when the net input is zero. “Leakiness” is a property observed in many applications, including the ones discussed in this paper.

We also assume that the probability of the 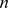-th node to transition from the active to the inactive state within 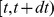 is given by 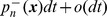, where the propensity function 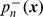 is given by
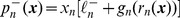
(2)for some nonnegative parameter 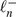 and a nonnegative function 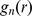. The term 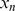 ensures that transition to the inactive state is possible only when the 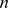-th node is active (i.e., when 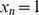), whereas the function 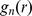 describes how the net input affects the probability of transition. On the other hand, when 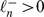, the parameter 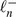 forces the node to be “leaky,” in the sense that it has a fixed propensity to transition from the active to the inactive state even when the net input is zero.

In general, the weights 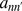 are used to determine the net input 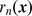 to node 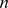. As a matter of fact, 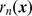 must not depend on 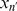 when 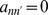. In particular, we set 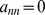, for every 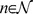, which implies that the nodes are not self-regulating. In some applications (such as the ones considered in this paper), we can set
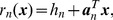
(3)where 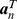 is the 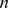-th row of the adjacency matrix 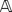 and 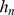 is a constant. In this case, 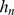 may represent the influence of external sources on the node (which we assume for simplicity to be fixed and known), whereas 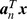 represents the influence of all active nodes in the network on the state of the 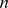-th node.


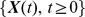
 is a Markov process. By assuming that all nodes in the network are initially inactive at time 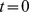, we can show that the probability distribution 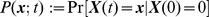 satisfies the master equation
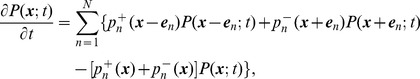
(4)for 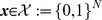, initialized with the Kronecker delta function 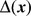 [i.e., 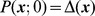], where 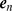 is the 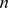-th column of the 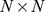 identity matrix. The model described by this equation is a continuous Boolean network model with state-dependent asynchronous node updating (more details can be found in Text S1). It can also be viewed as a special case of an interacting particle system (IPS) model [25] and it thus follows IPS-like dynamics. Unfortunately, solving this equation is a notoriously difficult task, especially when the number 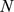 of nodes in the network is large. This is due to the fact that we need to calculate the probabilities 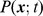, for 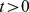, at every point 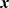 in the state space 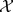, whose cardinality 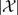 grows exponentially as a function of 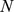, since 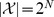.

### Coarse graining

We address the previous problem by employing a “coarse graining” procedure, similar to that suggested in [26] (see also [27]), which allows us to appreciably reduce the size of the state space while retaining key properties of the system under consideration. We assume that we can partition the population 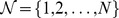 of all species in the network 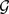 into 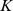 homogenous sub-populations 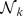, 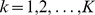, where 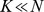. Due to the homogeneity of each sub-population, it may not be of particular interest to track the states of individual species in a given sub-population 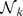. Instead, it may be sufficient to track the fraction 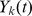 of active species in 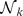, defined by
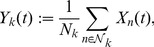
(5)where 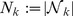. In this case, we may replace the original network with a smaller directed weighted network 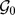 comprised of 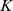 nodes from the set 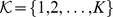 that represent the homogeneous sub-populations. We assume that, for every 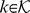, there exists a function 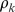 such that 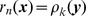, for all 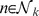, where 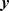 is a 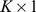 vector whose 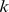-th element 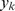 is given by 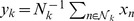. In this case, the stochastic process 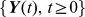 is also Markovian, governed by the following master equation (details in Text S1)
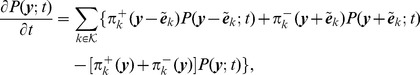
(6)initialized with the Kronecker delta function 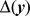 [i.e., 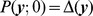], where 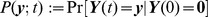 and 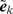 is the 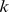-th column of the 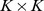 identity matrix multiplied by 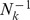. The new propensity functions are given by
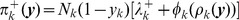
(7)
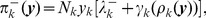
(8)where 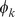, 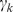, 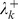, and 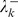 are such that, for every 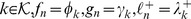, and 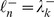, for 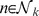. We refer to 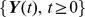 as the fractional activity process.

When the input to a node 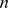 of the network is given by 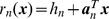, there is indeed a function 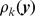 so that 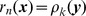, for every 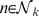. This function is given by 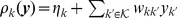, where 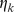 and 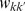 are such that 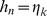, for every 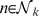, and 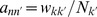, for every 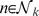, 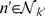 (details in Text S1). Note also that 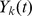 takes values in 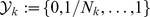. Therefore, the fractional activity process 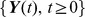 takes values in 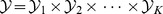. As a result, the state-space 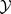 will be appreciably smaller than 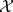, since 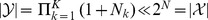, and solving the master equation of the fractional activity process will be easier than solving the master equation of the activity process.

### Macroscopic equations and LNA

We define the fractional “size” of the 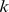-th sub-population as 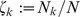. The thermodynamic limit is obtained by taking 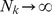, for every 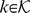, such that all 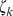
's remain fixed. In this case, 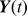 becomes a continuous random variable in the 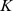-dimensional closed unit hypercube 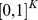. Furthermore, since 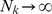, for every 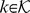, one might expect that the intrinsic noise at each node of the coarse network 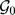 will be averaged out due to coarse graining. As a matter of fact, it can be shown that 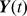 converges in distribution to the deterministic solution 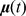 of the macroscopic differential equations
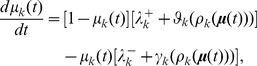
(9)for 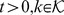, initialized by 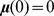. For simplicity of notation, we denote the thermodynamic limit by 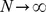.

If the macroscopic equations have a unique and stable fixed point 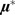 in the interior of the unit hypercube 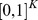, then for large enough but finite 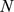, the linear noise approximation (LNA) method of van Kampen [11] allows us to approximate the fractional activity process 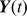 by adding correlated Gaussian noise 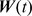 to the macroscopic solution 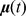. In this case,
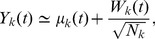
(10)for 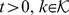, where, for each 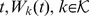, are zero-mean correlated Gaussian random variables with correlations 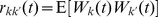 that satisfy a system of Lyapunov equations (details in Text S1). As a consequence, 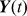 is approximated by a multivariate Gaussian random vector with mean 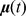 and covariance matrix 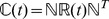, where 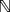 is a diagonal matrix with elements 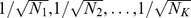, and 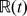 is the correlation matrix of random vector 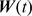.

### Potential energy landscape

We consider the probability distribution 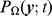 of the fractional activity process 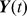 at time 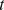, where we explicitly denote the dependence of this distribution on the network size 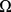. Let 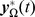 be a state in 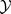 at which 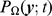 attains its (global) maximum value at time 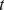 and define the function
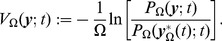
(11)Note that 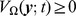 with equality if and only if 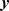 is a state at which 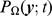 attains its (global) maximum value, known as a ground state. Moreover,
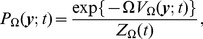
(12)for 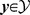, 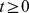, where 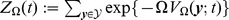. In this case, 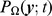 is a Boltzmann-Gibbs distribution with potential energy function 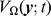 and partition function 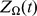. The (local or global) minima of 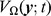 are associated with “potential wells” (basins of attraction) in the energy surface, which correspond to peaks in the probability distribution 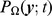. We may therefore view the fractional activity dynamics as fluctuations on a time-evolving potential energy landscape 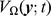 in the multidimensional state-space 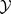, where downhill motions (towards the bottom of a potential well) are preferred with high probability, but random uphill motions can also occur with increasing probability as the population size decreases.

We are interested in the stationary potential energy 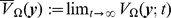, since its landscape remains fixed once the stochastic dynamics reach this point. At steady-state, the fractional activity dynamics simply perform a random walk on 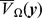. To compute 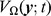 and 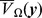, we solve the master equation of the fractional activity process numerically.

### Thermodynamic stability, robustness, and critical behavior

We characterize the stability, robustness, and critical properties of a LMN by studying its behavior when nodes are removed from the network. To do so, we introduce a number of quantities from classical thermodynamics which are used to describe the behavior of a physical system as its volume contracts or expands (see Text S1 for details). We focus our interest here at steady-state (irreducibility properties of the LMN model are discussed in Text S1). We define the internal potential energy by 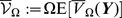, where 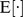 denotes expectation with respect to the stationary probability distribution 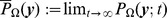. Moreover, we define the free potential energy by 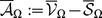, where 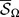 is the entropy of the network, given by 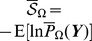. In thermodynamic terms, the free potential energy measures the portion of the energy, not accounted for by the energy of the most likely state, available in the LMN to do work at fixed size.

We also define the internal pressure 
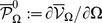
, pressure 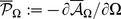, and bulk modulus 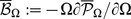. The internal pressure quantifies the rate of change in internal potential energy with respect to a change in the number of nodes, whereas the pressure quantifies the rate of change in free potential energy. Finally, the bulk modulus measures the network's resistance to changing pressure.

It turns out that 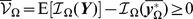, where 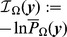 is the self-information of state 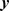 that quantifies the amount of information associated with the occurrence of state 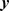 at steady-state. Therefore, and from an information-theoretic perspective, the internal potential energy of a LMN measures how far the self-information of the most likely state at steady-state is from the expected self-information of all network states (which is the entropy). Note that zero internal potential energy implies zero self-information for the most likely state. In this case, the LMN will be at the most likely state with probability one. As a consequence, we may consider the internal potential energy as a thermodynamic measure of the “stability” of a particular ground state of the potential energy landscape with smaller values indicating increasing stability of that state.

We can use the pressure as a measure of (thermodynamic) robustness of a LMN with respect to the network size 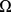. We say that a LMN is robust against variations in network size if there is no appreciable change in pressure when adding or removing nodes. Therefore, a LMN is robust if the derivative 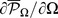 of the pressure or the bulk modulus is small (especially at small network sizes). This implies that a robust network must significantly resist changes in pressure.

Finally, we can use the bulk modulus 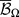 to detect network sizes at which a LMN exhibits critical behavior. As a matter of fact, it is well-known that an intensive thermodynamic quantity, such as the pressure, may experience a sharp discontinuity when another thermodynamic variable, such as the network size, varies past a critical value. If the pressure 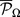 of a LMN experiences such a discontinuity as the size 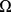 varies past a critical value 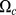, then 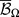 will effectively capture this discontinuity by a pulse at 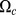, thus indicating that the network experiences phase transition at 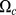.

## Results

### SISa and NN models

We explored our methods by considering two examples: a stochastic version of a one-dimensional SISa model of Methicillin resistant *staphylococcus aureus* (MRSA) infection [28], [29] with one homogenous population of 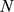 individuals, and a two-dimensional stochastic neural network (NN) model with two homogeneous populations of an equal number 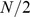 of *excitatory* and *inhibitory* neurons [10] (details in Text S1). With the first example, we were able to demonstrate for the first time that avalanching can also occur in epidemiology, even when simple models are used. In the second example, we show that the LNA method is not an appropriate tool for explaining the emergence of bursting and avalanching in neural network models. Despite a difference in dimensionality and their functional form, the two examples produce surprisingly similar results. The code, written in 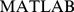, used to produce the results can be freely downloaded from www.cis.jhu.edu/~goutsias/CSS%20lab/software.html.

### Thermodynamic analysis reveals critical behavior in LMNs

**Figure 1:**
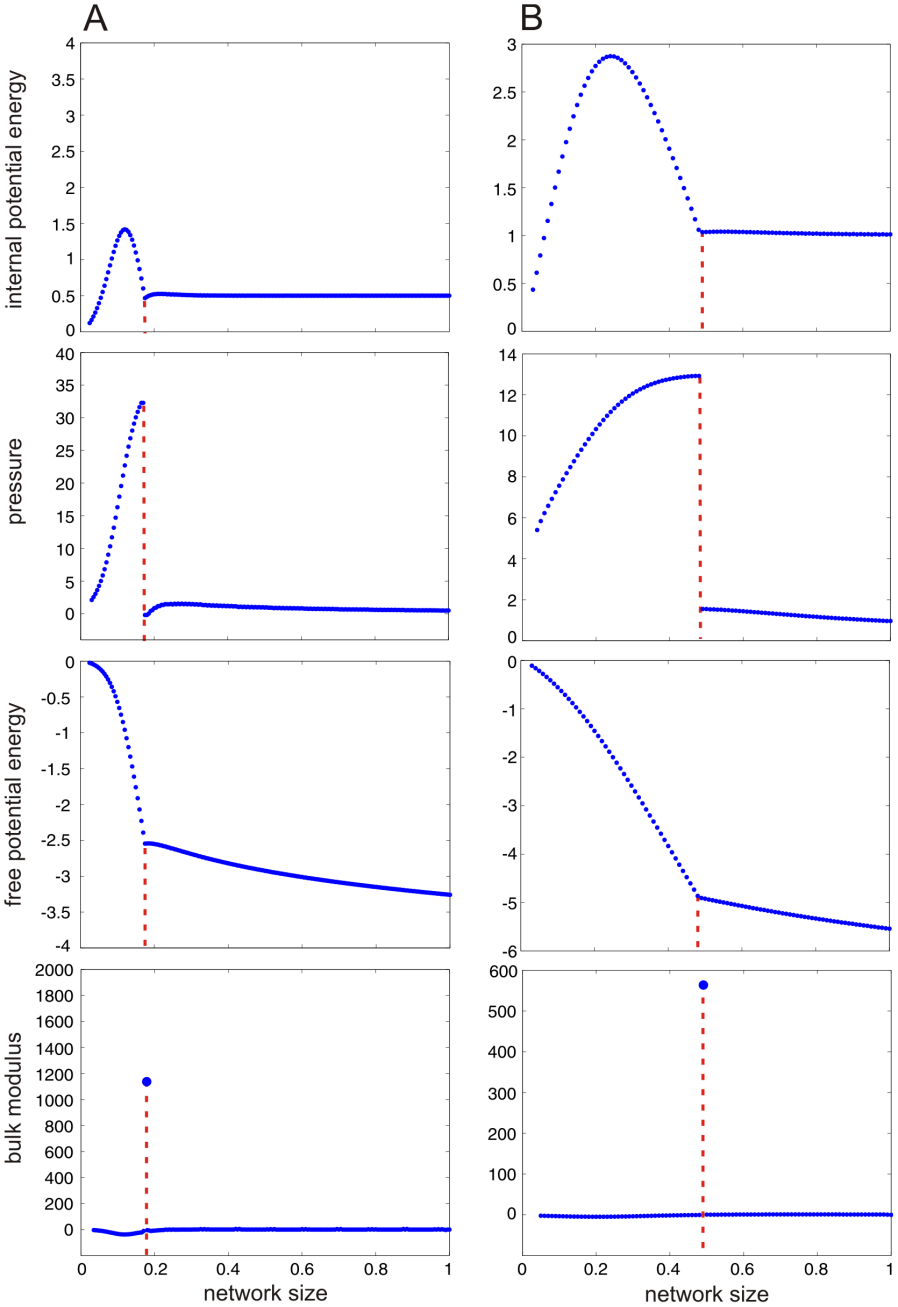
Thermodynamic behavior of two LMN models. Thermodynamic quantities computed for the SISa model, in (A), and the NN model, in (B), shown as a function of network size 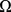. The red dashed lines mark the critical network size: 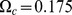 for the SISa model and 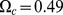 for the NN model. The results for the two models are qualitatively identical, despite the fact that their state spaces are of different dimensionality.

For each model, we computed the probability distribution 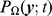 and the potential energy landscape 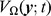, defined by Eq. (11), parameterized by the network size 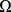, where 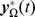 is a state at which 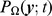 attains its (global) maximum. Figure 1 depicts four computed thermodynamic quantities as a function of 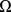, whereas, the supporting Figures S1, S2, S3, S4 depict movies of the dynamic evolutions of the stationary potential energy landscapes and probability distributions with respect to decreasing 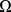. The results for the two models are qualitatively identical, despite the fact that the dimensionality of their state spaces are different. Note that the internal and free potential energy plots exhibit a deflection point at network size 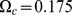 (population size 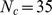), for the SISa model, and at 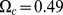 (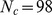), for the NN model, revealing critical behavior. This is also evident from the pressure, which experiences a discontinuity at 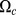 and produces a spike in the bulk modulus. On the other hand, the values of the bulk modulus are very close to zero at all other network sizes. For this reason, we can conclude that both models are *robust* with respect to network size (and hence to variations in the strength of intrinsic noise) away from the critical value 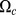.

**Figure 2:**
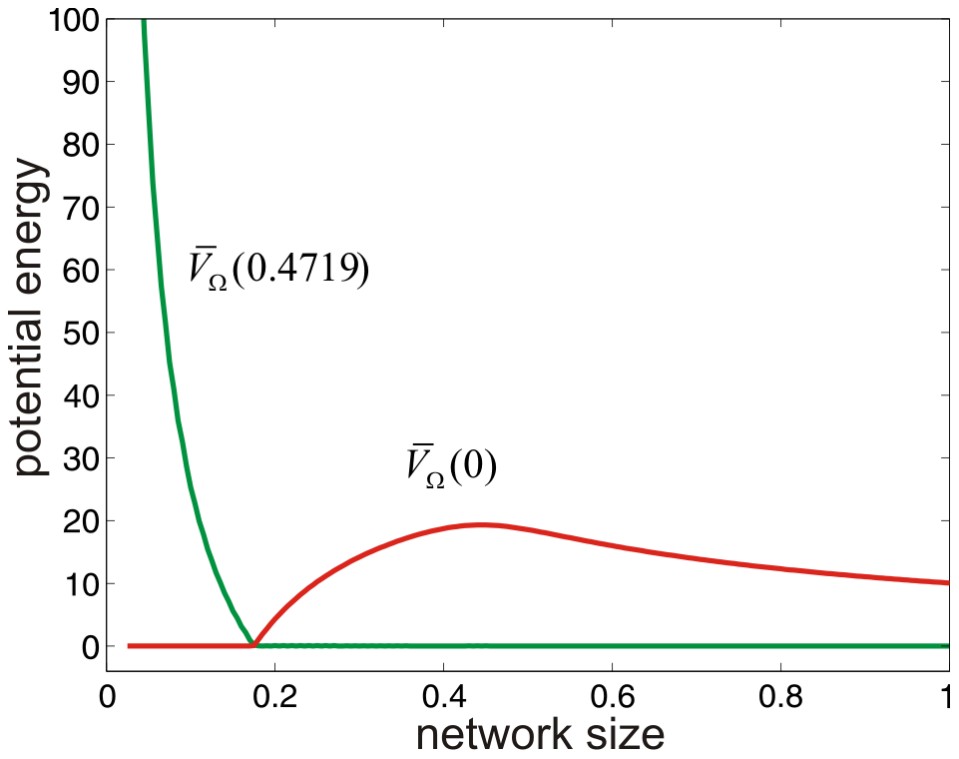
Critical behavior in the SISa model. The red curve depicts the value 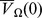 of the potential energy landscape as a function of 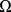, whereas the green curve depicts the value 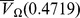. The two curves intersect at 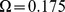, which is a critical network size.

What is the underlying cause of this critical behavior? The previous results suggest that the slope of the self-information support curve 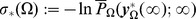 will experience a discontinuity at the critical size 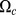 and a large curvature at that size. This is a consequence of the fact that 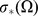 equals the pressure (see Text S1). Hence, loss of network robustness near 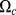 indicates that there is a change in the ground state (global minimum) of the stationary potential energy landscape at 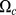. The movies depicted in Figures S1 & S3 corroborate the validity of this point. In particular, Figure 2 confirms that critical behavior in the SISa model is caused by the ground state of the potential energy landscape changing from the fixed point 0.4719 of the macroscopic equation [Eq. (S83) in Text S1] to the origin 0 of the state-space as the network size decreases past the critical value 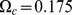. Likewise, critical behavior in the MM model is caused by the ground state changing from 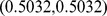 to 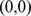 at 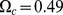.

### LNA fails to accurately predict rare large deviation excursions to the active and inactive states

**Figure 3:**
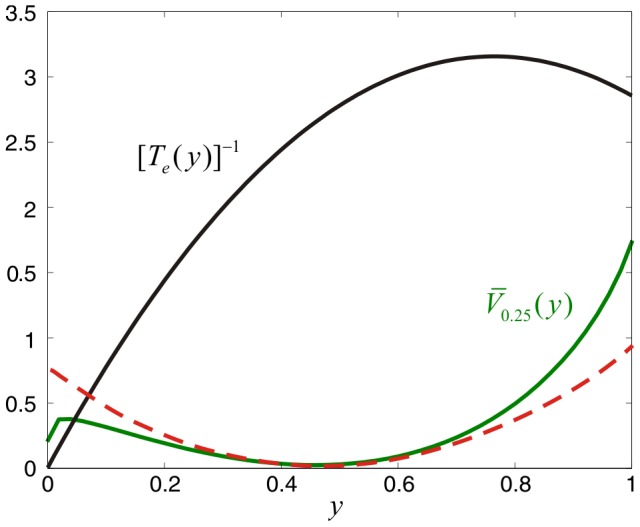
The LNA method fails to accurately predict rare deviations in the SISa model. The green curve depicts the stationary potential energy landscape 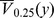, when 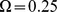 (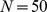), superimposed over the potential energy landscape predicted by the LNA method (dashed red curve). The black curve depicts the inverse mean escape time 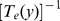 from a state 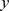 as a function of 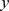. If the fractional activity process moves to a state at the left end tail of the potential energy landscape, it may stay there for an appreciable amount of time before returning back to the macroscopic ground state, whereas, if the fractional activity process moves to a state at the right end tail, it may quickly return back to the macroscopic ground state. This behavior is not well-predicted by LNA.

Figure S2 demonstrates that, for large 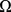, the LNA method provides a reasonable approximation to the stationary probability distribution of the SISa model. This observation however becomes questionable upon closer examination of the potential energy landscape dynamics depicted in Figure S1. Although the LNA potential energy landscape approximates well the true energy landscape over an appreciable region around the macroscopic ground state 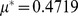, which accounts for about 99.8% of probability mass, there are substantial differences at the left and right tails of the landscape. These tails characterize *rare* large deviations from the macroscopic ground state and do not conform to the parabolic shape predicted by LNA. As a matter of fact, state values smaller than 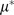 reside over a lower and flatter landscape than the one predicted by LNA, whereas state values larger than 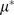 reside over a higher and steeper landscape. As a consequence, if the fractional activity process moves to a state at the left end tail of the potential energy landscape (i.e., close to the *inactive* state 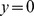), it may stay there for an appreciable amount of time before returning back to the macroscopic ground state. On the other hand, if the fractional activity process moves to a state at the right end tail (i.e., close to the *active* state 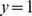), it may quickly return back to the macroscopic ground state. This behavior, which is not well-predicted by LNA, is corroborated by Figure 3, which shows the actual stationary potential energy landscape as a function of 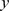 when 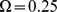 (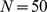), the potential energy landscape predicted by the LNA method, and the inverse mean escape time 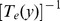 from a state 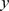 [computed from Eqs. (S73), (S81) & (S82) in Text S1]. Similar remarks hold for the NN model.

### Stability of inactive state is directly linked to strength of intrinsic noise

As the network size 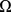 decreases towards the critical value 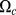, the approximation produced by the LNA method begins to break down, due to the emergence of a second well in the potential energy landscape located at the inactive state 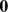 (see the movies depicted in Figures S1 & S3). This potential well becomes increasingly dominant, as compared to the well located at 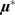. Since the ground state of the potential energy landscape transitions from 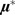 to 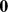 at the critical size 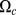 and remains at 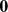 for all 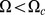, we expect the inactive state to be the most stable state at subcritical network sizes.

The internal potential energy remains fixed at supercritical network sizes; see Figure 1. This is predicted by Eq. (S61) in Text S1 and the fact that the LNA method provides a good approximation to the solution of the master equation at supercritical sizes (note that 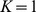 for the SISa model and 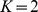 for the NN model). At subcritical sizes, the internal potential energy monotonically increases initially to a maximum value at some network size 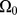 (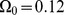 for the SISa model and 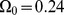 for the NN model) and subsequently monotonically decreases to zero. As a consequence, the internal pressure (which is the derivative of the internal potential energy with respect to 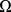) is negative for 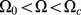 and positive for 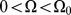. Positive internal pressure (decreasing internal potential energy) signifies the fact that removing nodes (individuals or neurons) from the network results in decreasing the distance between the self-information of the most likely state (i.e., the amount of information associated with the occurrence of the inactive state) from the average self-information of all states and thus increasing the stability of this state (details can be found in Text S1). As a consequence, and for network sizes below 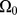, increasing levels of intrinsic noise result in increasing the stability of the inactive state

### Emergence of the noise-induced mode leads to bursting

**Figure 4:**
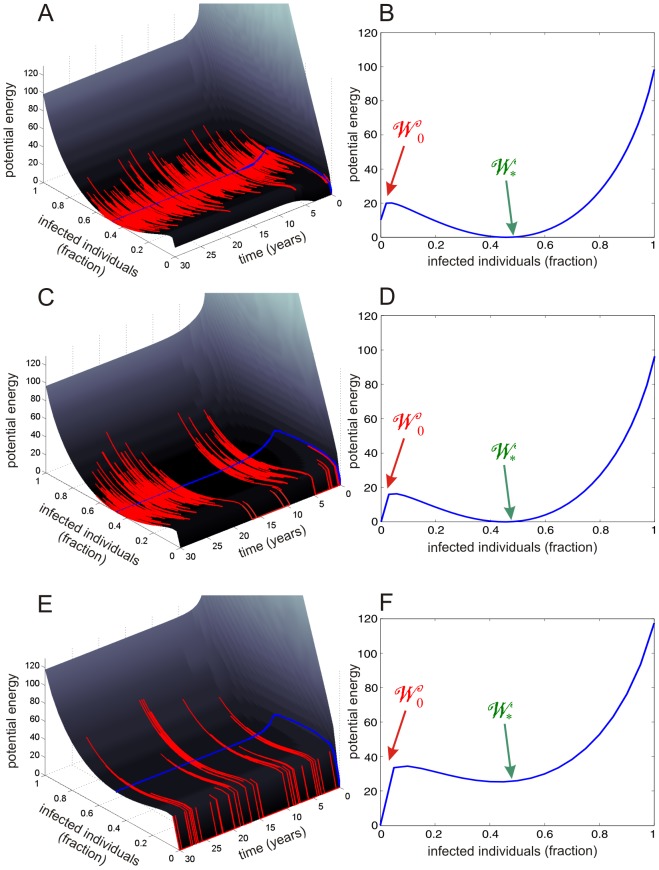
Emergence of noise-induced ground state and bursting in the SISa model. 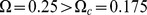
 (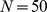) in (A, B), 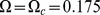 (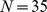) in (C, D), and 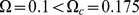 (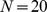) in (E, F). The left column depicts a single stochastic trajectory of the activity process (in red) along with the corresponding macroscopic solution (in blue), superimposed on the potential energy landscape. The right column depicts the corresponding stationary potential energy landscapes. See Text S1 for a discussion about the long time scales involved.

To investigate the emergence of bursting in the SISa model, we depict in the first column of Figure 4 realizations of the fractional activity process (red lines) and the macroscopic dynamics (blue lines), superimposed over the potential energy landscape, for three network sizes, namely 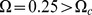 (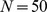) in A, 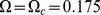 (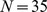) in C, and 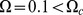 (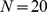) in E. Moreover, we depict in the second column of Figure 4 the corresponding stationary potential energy landscapes.

The stationary energy landscape depicted in Figure 4*B* exhibits two potential wells. A shallow and narrow well 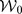 located at 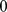 and a relatively deep and wide well 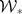 located at the stable fixed point 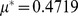 of the macroscopic equation. Transitions from 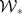 into 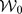 are dubious, since such transitions require appreciable stochastic deviations, which are not likely to take place. On the other hand, transitions from 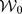 to 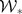 are easier, requiring smaller stochastic fluctuations (mean escape time from 0 is 200 days). In this case, the fraction of infected individuals will fluctuate in a Gaussian-like manner around 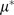, although it may sometimes become zero for a relatively short period of time; see Figure 4*A*.

Figures 4*D & 4F* indicate that, as the network size decreases, the first potential well 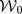 becomes deeper and wider, whereas the second well 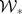 becomes shallower and eventually disappears; see also the movie depicted in Figure S1. When 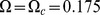, the two potential wells achieve the same depth. In this case, the fractional activity processes may remain inside 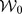 longer than before, since transitions from 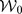 into 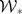 become more difficult (mean escape time from 0 is now 286 days). As a consequence, the fraction of infected individuals will fluctuate in a Gaussian-like manner around 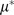 as before, although it may now become zero for a longer period of time; see Figure 4*C*.

**Figure 5:**
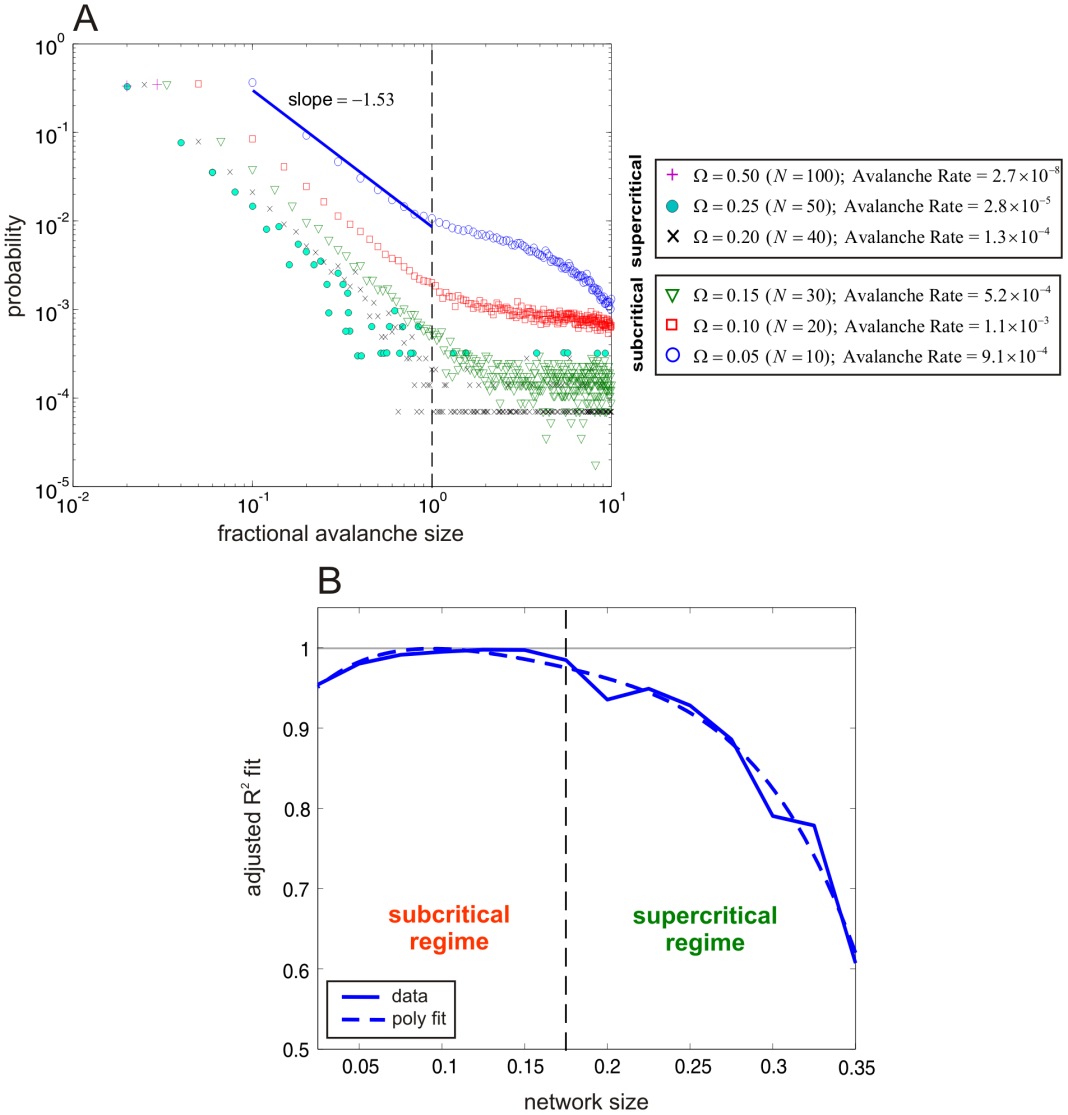
Avalanche statistics reveal scale-free behavior at subcritical network sizes for the SISa model. (A) Log-log plot of estimated probability distributions of the fractional avalanche size for various network sizes 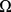. The cases corresponding to subcritical network sizes below 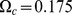 exhibit high rates of avalanching with fractional avalanche size distributions characterized by scale-free behavior for sizes smaller than 1. The cases corresponding to the supercritical network sizes exhibit increasingly lower rates of avalanching and gradual break-down of scale-free behavior. The blue line indicates a linear square fit of the data when 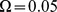. (B) Adjusted 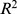 values (solid blue curve) of the goodness of fit of a linear regression of a portion (below 1) of the log-log probability distribution of fractional avalanche size computed at discrete network sizes 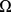. 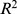 values close to one indicate scale-free (linear) behavior. Standard 4-th order polynomial fit of the computed 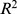 values produced a smoother curve (dashed blue curve). The scale-free property of avalanching is characteristic to network sizes close or below the critical size 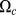 (dashed black line) and disappears gradually as 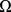 increases away from the critical size, as indicated by the decreasing 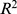 values.

On the other hand, Figure 4*F* indicates that, when 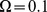, the potential well 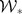 becomes extremely shallow. In this case, the fractional activity process will spend most time within 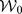 with infrequent and very short excursions outside this well (mean escape time from 0 is 500 days). As a consequence, the fraction of infected individuals will mostly be zero with occasional and brief switching to nonzero values. This bursting behavior is clear from figure 5*E* and is expected in the SISa model since, in a hospital setting or in a swine herd, one often speaks of unpredictable “outbreaks” of an infection, such as MRSA. The deterministic SISa model is fundamentally incapable of predicting such complex behavior. Similar remarks apply for the NN model; see the supporting Figure S5.

### Avalanche formation becomes a rare event at supercritical network sizes

**Figure 6:**
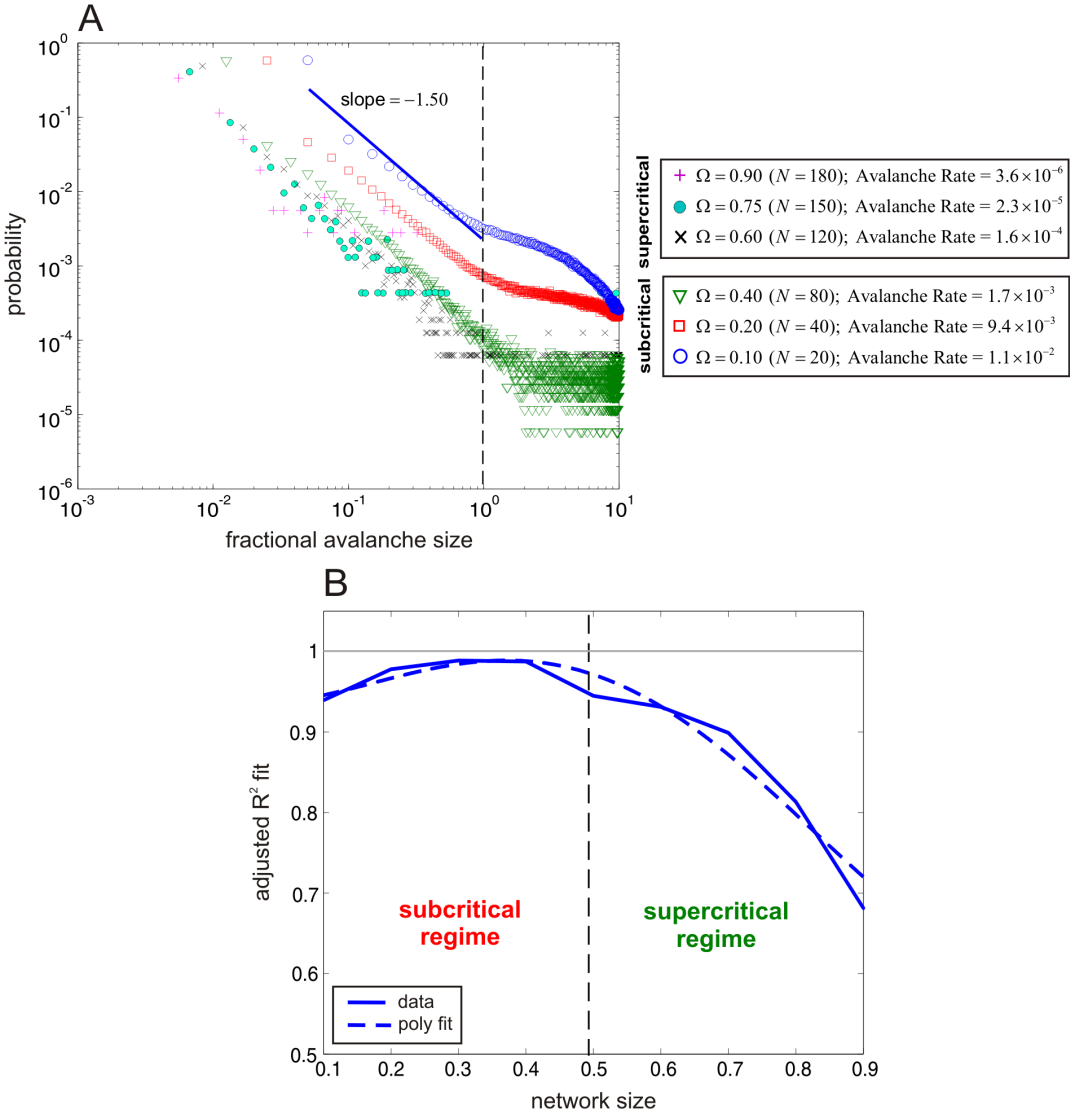
Avalanche statistics reveal scale-free behavior at subcritical network sizes for the NN model. (A) Log-log plot of estimated probability distributions of the fractional avalanche size for various network sizes 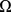. The cases corresponding to subcritical network sizes below 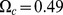 exhibit high rates of avalanching with fractional avalanche size distributions characterized by scale-free behavior for sizes smaller than 1. The cases corresponding to the supercritical network sizes exhibit increasingly lower rates of avalanching and gradual break-down of scale-free behavior. The blue line indicates a linear square fit of the data when 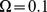. (B) Adjusted 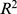 values (solid blue curve) of the goodness of fit of a linear regression of a portion (below 1) of the log-log probability distribution of fractional avalanche size computed at discrete network sizes 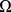.

Because bursting occurs primarily at steady-state (see Figure 4), we computed avalanche statistics from a single trajectory of the fractional activity process obtained from a long sample of this process. This helped us reduce the computational effort required when calculating avalanche statistics from multiple runs. We simulated the SISa model using the Gillespie algorithm for a period of 300,000 years and used an avalanching threshold 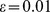 to compute the presence of an avalanche (details in Text S1). This allowed us to characterize the SISa model as being active if at least 1 out of 100 individuals was infected. In Figure 5*A*, we depict log-log plots of the estimated probability distributions of the fractional avalanche size, for sizes between 0.01 and 10 and for various choices of 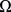. We also depict the rate of avalanche formation for each case, calculated as the number of avalanches that occurred per day. For the three subcritical network sizes below 0.175, the distributions exhibit scale-free behavior (i.e., the log-log plots are linear) for fractional avalanche sizes below 1 (i.e., when the number of infections that occur during an avalanche is at most 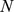). This observation is clearly corroborated by the results depicted in Figure 5*B*. On the other hand, for the three supercritical network sizes above 0.175, we observe increasingly lower rates of avalanching, indicating that avalanche formation becomes eventually a rare event as 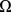 increases. Moreover, this is accompanied with a loss of the scale-free behavior of the size distribution, as it is clearly indicated by Figure 5*B*. We obtained similar results for the NN model; see Figures 6A & 6B.

### Predicted near-critical avalanche behavior concurs with real experimental data

It has been argued in the literature that the empirical observation of a power law in a statistical distribution is not sufficient to establish criticality [30], [31]. This has been particularly scrutinized in the case of neural networks and specifically in studying bursting [32]–[34]. Although our analysis clearly shows that a LMN model will experience critical behavior as its size decreases from the supercritical to the subcritical regime, quantified by an observed abrupt behavior in the value of the bulk modulus, an important question that arises at this point is whether this prediction concurs with real experimental data. By employing a method known as avalanche shape collapse [7], [34], we can demonstrate that our thermodynamic predictions lead to near-critical avalanching behavior that can be confirmed by real experimental data.

Avalanche shape collapse is used to demonstrate whether the power laws observed in neural bursting is the result of a mechanism operating near criticality. The basic idea behind this method is that the distributions of some variables of interest in a neural system near criticality are characterized by power laws. Given avalanche data, we can organize avalanches into groups of the same duration 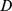. We can then draw a log-log plot of the average size 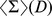 of observed avalanches of duration 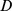 as a function of 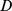 and expect that a linear trend will emerge with slope 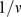 [i.e., we expect that the distribution of 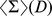 will be proportional to 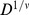]. Note that, in order to compare our results with the ones obtained in [7], we employ here the definitions for avalanche duration and size obtained by partitioning time into bins of equal duration 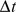; see Text S1. Within each avalanche group of size 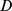, we can compute the *avalanche shape* by plotting the average number 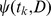 of neurons firing within the time interval 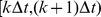 as a function of 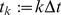, for 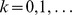. Note that right before and right after an avalanche there are no neurons firing. Therefore, the avalanche shape describes the average pattern of non-zero neuron firings between these two lulls in activity.

If observed avalanche cascades are truly critical, then we expect that the computed avalanche shapes will be self-similar, in which case [7]
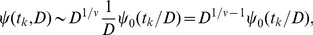
(13)where 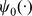 is a scaling function that does not depend on 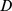. In this case,
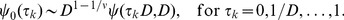
(14)By testing whether available experimental data confirm these conditions, we can increase our confidence that the data come from a neural network which operates near criticality. We can accomplish this by shape collapse: we multiply each avalanche shape 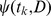 with 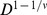 and rescale time to be between 0 and 1 by dividing 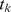 with 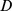.

**Figure 7:**
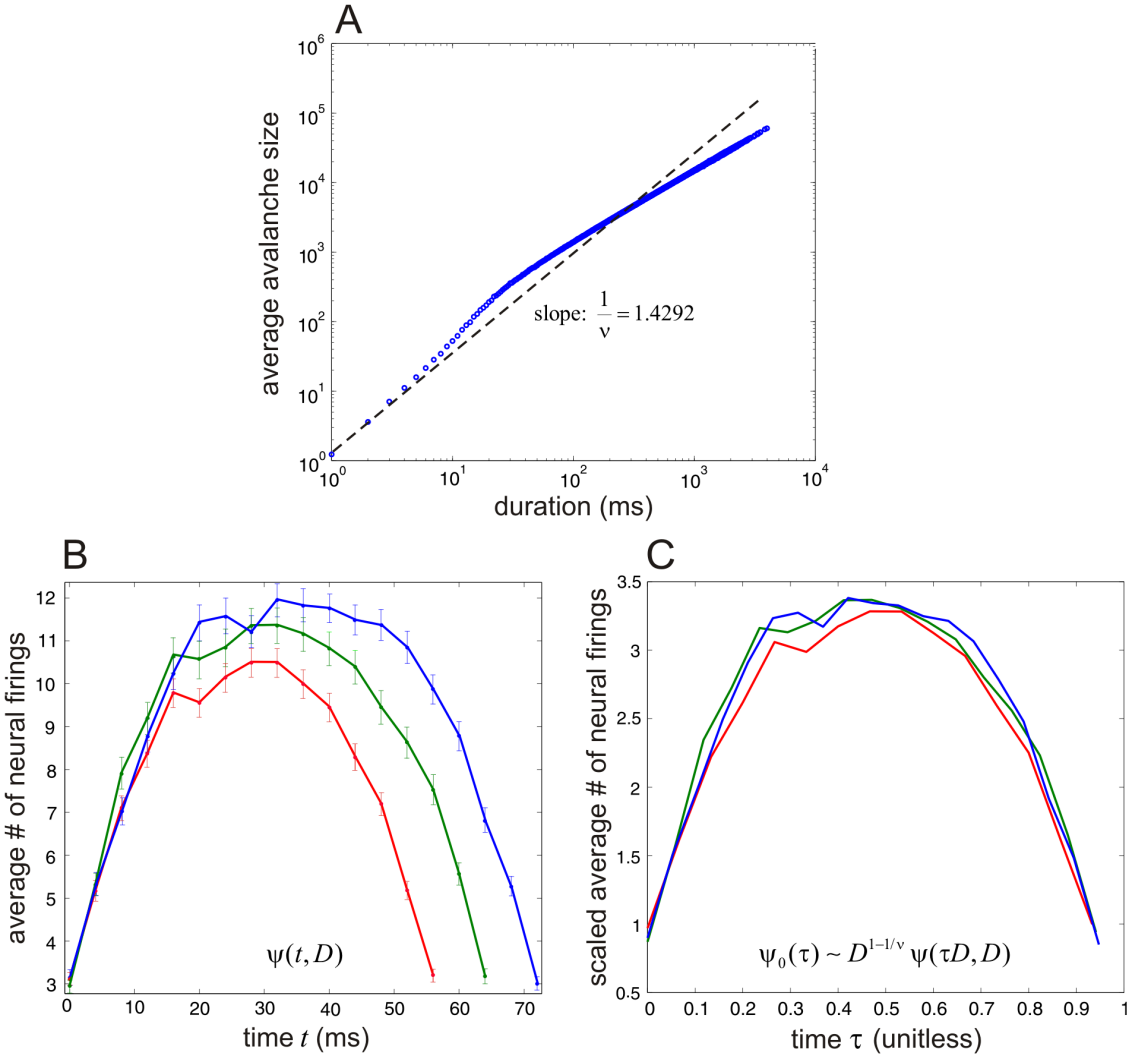
Avalanche shape collapse concurs with real experimental data and confirms predicted near-critical avalanche behavior in the NN model. (A) A log-log plot of the average avalanche size of simulated avalanches as a function of avalanche duration in a LMN neural network model with 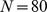 neurons. A linear fit of the plot reveals a slope of 1.4292. These results agree well with the corresponding results depicted in Figure 2 of [7] obtained from real experimental data. (B) Avalanche shapes obtained for three different durations: 60 ms (red curve), 68 ms (green curve), and 76 ms (blue curve). The error bars quantify the standard error occurred by estimating the average number of neural firings using Monte Carlo. (C) Shape collapse plots corresponding to the curves in (B). The results closely match the results depicted in Figure 3 of [7] obtained from real experimental data and confirms the critical behavior predicted by thermodynamic analysis.

Avalanche shape collapse has been recently applied on real experimental data and has confirmed Eq. (14) – see Figure 3 in [7]. To investigate whether our LMN neural network model concurs with these results, we have simulated our LMN neural network model with 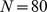 neurons (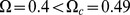) for a period of 80,000,000 ms (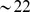 hours). We then implemented the shape collapse method by partitioning time into 20,000,000 bins of 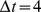 ms each. The results depicted in Figure 7 agree with the corresponding results depicted in Figures 2 & 3 of [7]. Our model captures well the inverted parabolic nature of avalanche shapes observed in real experimental data and our simulation results confirm that the dynamics of our model undergo a shape collapse similar to that observed in [7]. As a consequence, our *in silico* experiment provides a conclusive argument that our model operates near criticality, confirming the validity of our thermodynamic analysis and further supporting the applicability of the proposed modeling and analysis approach to avalanching.

### External influences affect avalanching

The previously discussed results for the SISa model are based on setting 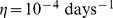. This parameter quantifies the influence of extrinsic factors (other than direct transmission from other infected individuals) on the rate of infection. We therefore investigated the effect of 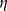 on bursting.

The mean escape time from the inactive state depends inversely proportional on the network size 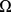 and parameter 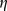. This implies that, for a fixed value of 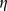, the stability of the inactive state increases for decreasing 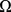, in agreement with our previous discussion.

For fixed 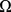, 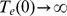, as 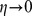, and moving away from the inactive state becomes increasingly difficult. When 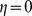, the SISa model reduces to the standard SIS model of epidemiology, which enjoys far simpler dynamics: infections will always die out and never appear again, since 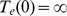. As a consequence, the stationary probability distribution of the SIS model assigns all probability mass to the inactive state. On the other hand, 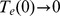, as 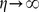, which implies that, for sufficiently large 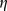, the SISa model will be moving away from the inactive state almost instantaneously.

**Figure 8:**
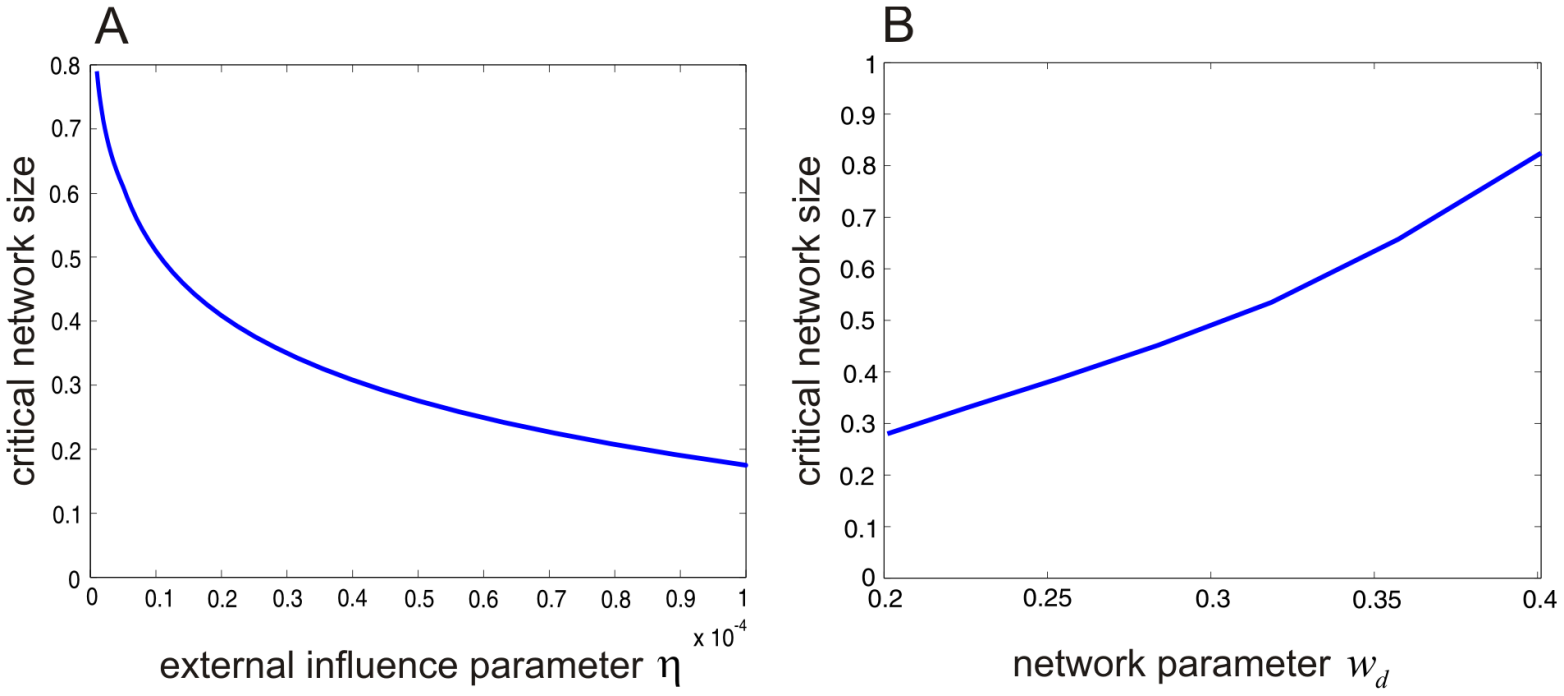
Dependence of critical network size on model parameters. (A) Critical network size 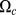 of the SISa model as a function of the external influence parameter 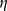. (B) Critical network size 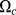 of the NN model as a function of parameter 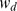.

Figure 8*A* depicts a plot of the critical network size 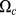 as a function of 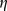, for 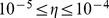. Clearly, decreasing 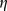 increases the value of 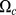. In particular, 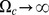, as 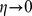. As a consequence, and for sufficiently small values of 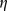, 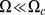, which implies that no avalanching is expected since the network will be operating in the subcritical regime far away from the critical point. This can also be explained by considering the fact that, for very small values of 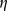, infection from sources other than infected individuals will be so small that the state of zero infective individuals will have such high probability that excursions from the origin (and thus avalanches) will be rare events.

Similarly, increasing 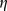 decreases 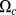. In particular, 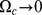, as 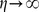. This implies that, for sufficiently large values of 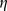, 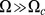, which implies that no avalanching is expected since the network will be operating in the supercritical regime far away from the critical point. This can also be explained by considering the fact that, for very large values of 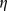, infection from sources other than infected individuals will be so prevalent that the state of zero infective individuals will have such low probability that excursions from the origin (and thus avalanches) will be rare events.

For the values of 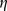 encountered in practice, we may have that 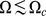. This implies that avalanching will be prevalent since the network will be operating in the subcritical regime close to the critical point. Similar results have been obtained for the NN model (data not shown).

### Balanced feed-forward structure is not necessary for avalanching in NNs

In a previous work [10], analysis of the NN model using the LNA method led to the conclusion that for a NN to exhibit bursting it is required that 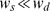, where 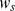, 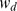 are two appropriately defined parameters (details about these parameters can be found in Text S1). When 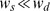, the neural network is *balanced*, in the sense that excitation is very close to inhibition. Moreover, it has been shown that, when the LNA method is valid, fluctuations in the average difference 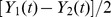 of the fractional activity processes of the excitatory and inhibitory neurons feed-forward into the evolution of the average sum 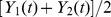. It was then argued that a balanced feed-forward (BFF) structure is necessary for avalanching in relatively large NNs and that this is achieved through amplification of low levels of intrinsic noise.

Our thermodynamic analysis demonstrates that bursting could be a noise-induced phenomenon that cannot be characterized by the LNA method. This is due to the fact that, at supercritical network sizes, the LNA method may not sufficiently approximate the potential energy landscape in a neighborhood of the inactive state, whereas the method breaks down completely at subcritical network sizes. As a matter of fact, the supporting Figure S6 shows that LNA produces a poor approximation to the potential energy landscape close to 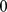 for the model considered in [10]. This is not surprising, since the LNA method always results in a negligible probability for the activity process to reach the inactive state 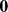 [35], which is a predicament that fundamentally contradicts the very basic and experimentally observed nature of avalanching.

It turns out that the BFF condition is not necessary for bursting in NNs. Instead, we have argued that bursting can be attributed to the gradual formation of the noise-induced mode at 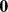 with decreasing network size.

To further confirm this point, note that BFF behavior is controlled by 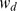 when 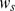 is held fixed [10]. With 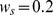, the analysis in [10] implies that the NN model will exhibit bursting only when 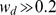. However, our results show that this is also true when 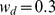; see Figure 6. In Figure 8*B*, we depict the computed critical system size 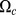 for a fixed value 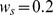 as a function of 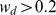. This result demonstrates that, increasing the value of 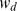 increases the critical network size. Therefore, for a NN with large 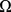 to exhibit bursting it is required that the value of 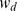 be sufficiently larger than the value of 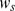. This implies that the NN must be balanced. Although the feed-forward condition is not necessary for bursting, it ensures that, in large NNs, the noise induced mode at 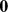 remains stable.

## Discussion

In this paper, energy landscape theory, combined with thermodynamic analysis, has led to a powerful methodology for the analysis of Markovian networks. By introducing leaky Markovian networks, we developed an *in silico* approach for understanding the origins of bursting. We have quantified topographic deformations of the energy landscape as a function of network size and showed that bursting is a complex behavior caused by the emergence of noise-induced modes and reallocation of ground states. This led to a novel view of avalanching as a complex behavior that dominates system dynamics at near-critical or subcritical network sizes caused by appreciable levels of intrinsic noise. Future improvements in computer hardware and software will allow our methods to be used in more complicated problems than the ones considered here in an effort to theoretically understand and experimentally evaluate bursting as well as other complex phenomena.

The main objective of our work is to demonstrate that intrinsic noise in complex networks of interacting elements can induce critical behavior leading to avalanching. The strength of intrinsic noise in a given network is inversely proportional to its size, quantified by the number of nodes in the network. As the network size decreases, a critical behavior in thermodynamic behavior is observed that leads to avalanching. This is contrary to what has been done so far in the literature, which is mainly focused on how extrinsic influences to a network lead to criticality and avalanching [5]. The methodology proposed in this paper provides a link between *statistical* and *dynamic* criticality, two well-known notions of critical behavior [5]. Our results clearly demonstrate that principles fundamental to statistical mechanics can be effectively used to study criticality in phenomena that are dynamic by nature, such as avalanching.

We should also note that living biological systems, such as neuronal networks, must necessarily exchange matter and energy with their surroundings (i.e., they are open thermodynamic systems) and, as such, they should operate away from thermodynamic equilibrium (see Text S1 for details). As a consequence, it is common to refer to a homeostatic state in biological systems as *non-equilibrium steady state* (NESS) to make explicit the fact that living biological systems in dynamic equilibrium are not in thermodynamic equilibrium.

**Figure 9:**
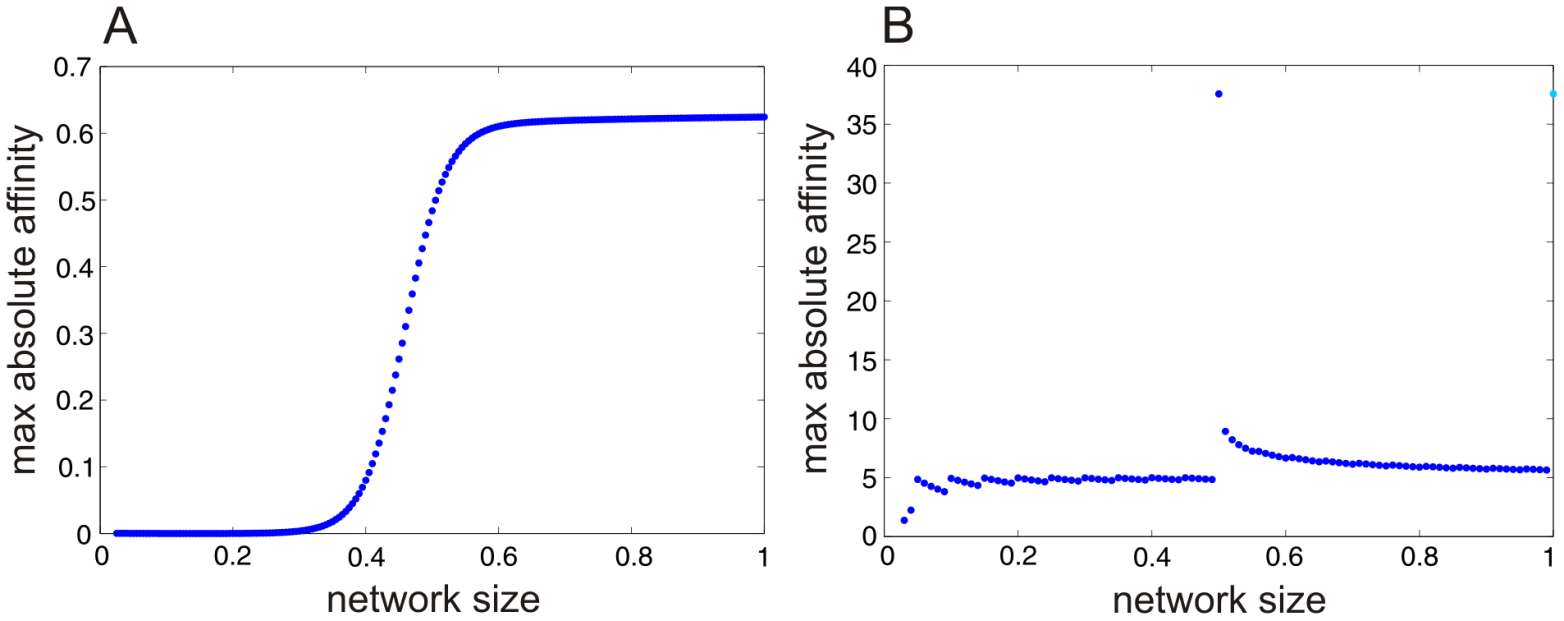
Dependence of thermodynamic equilibrium on network size. (A) The maximum absolute affinity in the SISa model reveals that the system is near thermodynamic equilibrium at network sizes 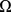 below 0.3. However, the system starts deviating from thermodynamic equilibrium as the network size increases beyond this value. (B) The maximum absolute affinity in the NN model reveals that the system is away from thermodynamic equilibrium at all network sizes considered.

In this paper, we utilize familiar tools (e.g., the Boltzmann-Gibbs distribution) originally introduced in statistical mechanics to study gasses at thermodynamic equilibrium. As a consequence, the reader may think that our analysis is limited to complex networks at equilibrium. Note however that our methods are based on a Boltzmann-Gibbs distribution defined in terms of the solution of the master equation, according to Eqs. (11) & (12), which are dynamic in nature. As a consequence, our analysis is not limited to equilibrium dynamics, because the master equation is capable of describing systems far from dynamic and thermodynamic equilibrium (for more details on these two types of equilibrium, the reader is referred to Text S1). As a matter of fact, in Figure 4, we depict results away from dynamic equilibrium. Moreover, in Figure 9, we depict the maximum absolute affinity 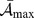 in the SISa and NN models, defined by Eq. (S72) in Text S1, for various system sizes 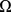. Figure 9A indicates that the SISa model is near thermodynamic equilibrium at network sizes below 0.3. However, this model moves away from thermodynamic equilibrium as 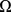 increases beyond this value. On the other hand, Figure 9B shows that the neural network model is away from thermodynamic equilibrium for all network sizes considered, as expected. Clearly, the tools suggested in this paper are fully capable of analyzing non-equilibrium systems and NESSs, such as those that arise in biology.

To conclude, we finally point out that, in some regions of the brain, the ratio of the excitatory to inhibitory neurons is closer to 4∶1 rather than the ratio of 1∶1 used in this paper. The main reason for our choice is to compare our results to the ones in [10], which are based on the 1∶1 ratio. Qualitatively, we do not expect that changing the ratio will alter our conclusions, and this was indeed confirmed by analyzing the leaky Markovian neural network model using a 4∶1 ratio (data not shown).

## Supporting Information

Figure S1**Stationary potential energy landscape of the SISa model.** Movie of the dynamic evolution, with respect to decreasing network size 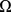, of the stationary potential energy landscape (blue solid curve). The red dashed curve represents the potential energy landscape predicted by the LNA method. The double headed arrow indicates the region of 99.8% probability predicted by the LNA method. In addition, the clip indicates when the LNA method produces a probability distribution that extends beyond the state space 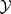.(GIF)

Figure S2**Stationary probability distribution of the SISa model.** Movie of the dynamic evolution, with respect to decreasing network size 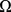, of the stationary probability distribution (blue solid curve). The red dashed curve represents the probability distribution predicted by the LNA method.(GIF)

Figure S3**Stationary potential energy landscape of the NN model.** Movie of the dynamic evolution, with respect to decreasing network size 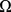, of the stationary potential energy landscape of the NN model.(GIF)

Figure S4**Stationary probability distribution of the NN model.** Movie of the dynamic evolution, with respect to decreasing network size 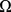, of the stationary probability distribution of the NN model.(GIF)

Figure S5**Noise-induced reallocation of the ground state of the stationary potential energy landscape in the NN model.** 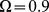 (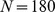) in (A), 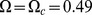 (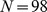) in (B), and 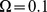 (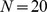) in (C). For network sizes above the critical value 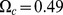, the ground state of the potential energy landscape is at the fixed point 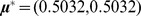, predicted by the macroscopic equations. As the network size 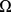 decreases from supercritical to subcritical values, the depth of the potential well located at 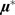 decreases, whereas a new potential well emerges, located at the inactive state 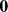, with increasing depth and width. For network sizes below the critical value, noise-induced deformation of the potential energy landscape results in a reallocation of the ground state from 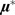 to the inactive state 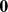. This type of “phase transition” demarcates the onset of avalanching.(TIF)

Figure S6**Failure of LNA to sufficiently approximate the stationary probability distribution in the NN model.** (A) The true stationary probability of the fractional activity process in the NN model considered in [10], with 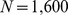, 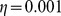, 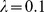, 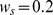, and 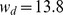. (B) The approximating stationary probability distribution obtained by the LNA method. Clearly, the LNA method provides a poor approximation to the actual probability distribution in this case. In particular, the true distribution depicted in (A) predicts a probability of 0.45 for the network to be at a state close to the inactive state 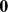 and a probability of 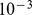 for the network to be at a state within a small neighborhood around the mode 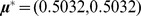, predicted by the macroscopic equations. On the other hand, the corresponding probabilities predicted by the sampled Gaussian distribution depicted in (B) are 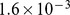 and 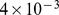.(TIF)

Text S1 **Additional mathematical and modeling details:** LMNs and Boolean networks, Markovianity of the fractional activity process, the linear noise approximation method applied to the LMN model, leakiness and irreducibility, definition of thermodynamic quantities and connections to thermodynamic stability, robustness and critical behavior, equilibrium and non-equilibrium behavior, relation between noise-induced modes, stochastic transition and bursting, new definition for avalanches, details on the epidemiological and neural network examples.
(PDF)
