# Supplementary figures and images for: Intrinsic Noise Induces Critical Behavior in Leaky Markovian Networks Leading to Avalanching
Source: PLoS Comput Biol. 2014 Jan 9;10(1):e1003411. doi: 10.1371/journal.pcbi.1003411 (PMC3886886; doi:10.1371/journal.pcbi.1003411)

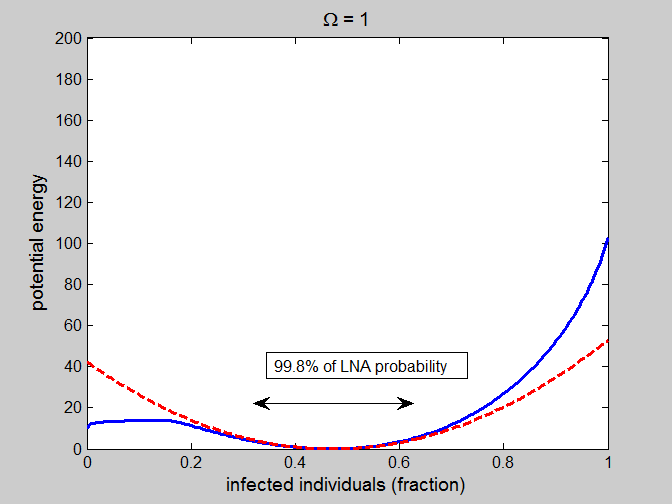

Supplement: Figure S1 — Stationary potential energy landscape of the SISa model. Movie of the dynamic evolution, with respect to decreasing network size , of the stationary potential energy landscape (blue solid curve). The red dashed curve represents the potential energy landscape predicted by the LNA method. The double headed arrow indicates the region of 99.8% probability predicted by the LNA method. In addition, the clip indicates when the LNA method produces a probability distribution that extends beyond the state space . (GIF) [file pcbi.1003411.s001.gif]

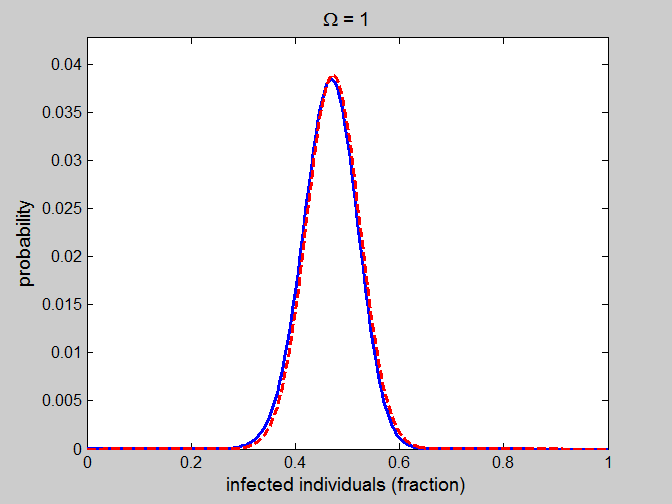

Supplement: Figure S2 — Stationary probability distribution of the SISa model. Movie of the dynamic evolution, with respect to decreasing network size , of the stationary probability distribution (blue solid curve). The red dashed curve represents the probability distribution predicted by the LNA method. (GIF) [file pcbi.1003411.s002.gif]

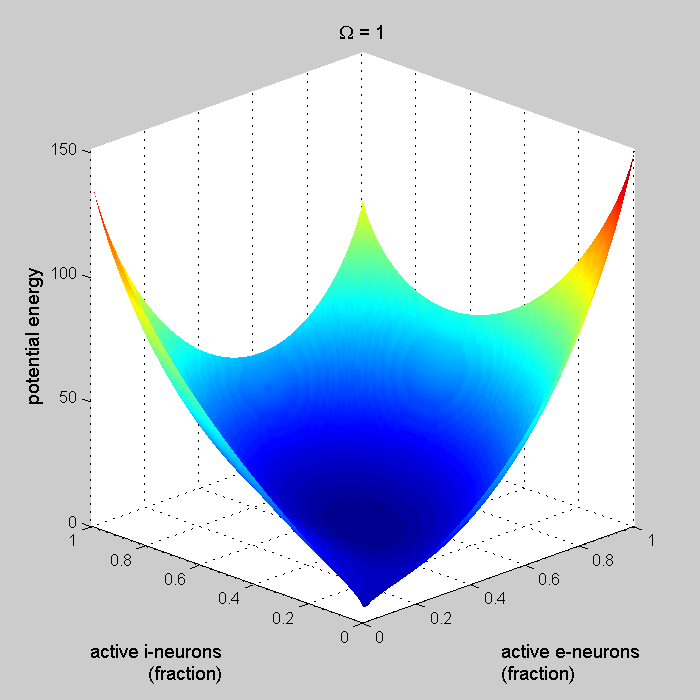

Supplement: Figure S3 — Stationary potential energy landscape of the NN model. Movie of the dynamic evolution, with respect to decreasing network size , of the stationary potential energy landscape of the NN model. (GIF) [file pcbi.1003411.s003.gif]

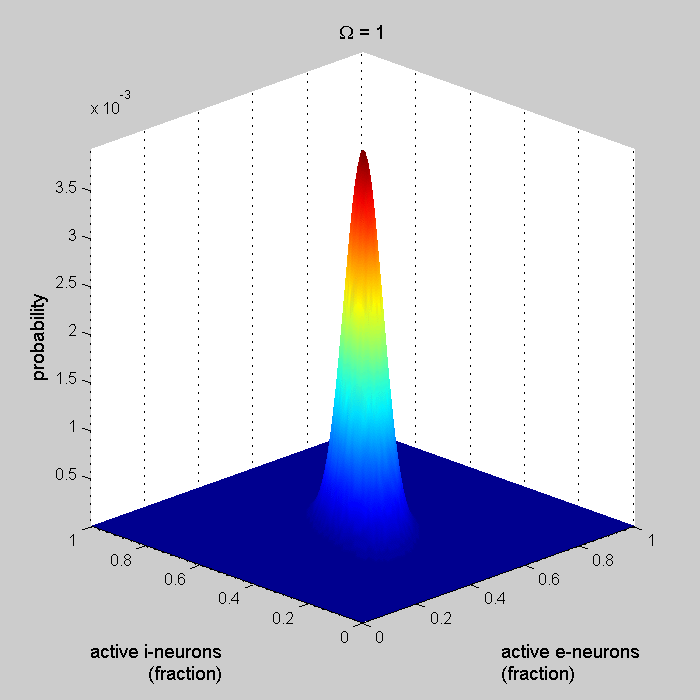

Supplement: Figure S4 — Stationary probability distribution of the NN model. Movie of the dynamic evolution, with respect to decreasing network size , of the stationary probability distribution of the NN model. (GIF) [file pcbi.1003411.s004.gif]

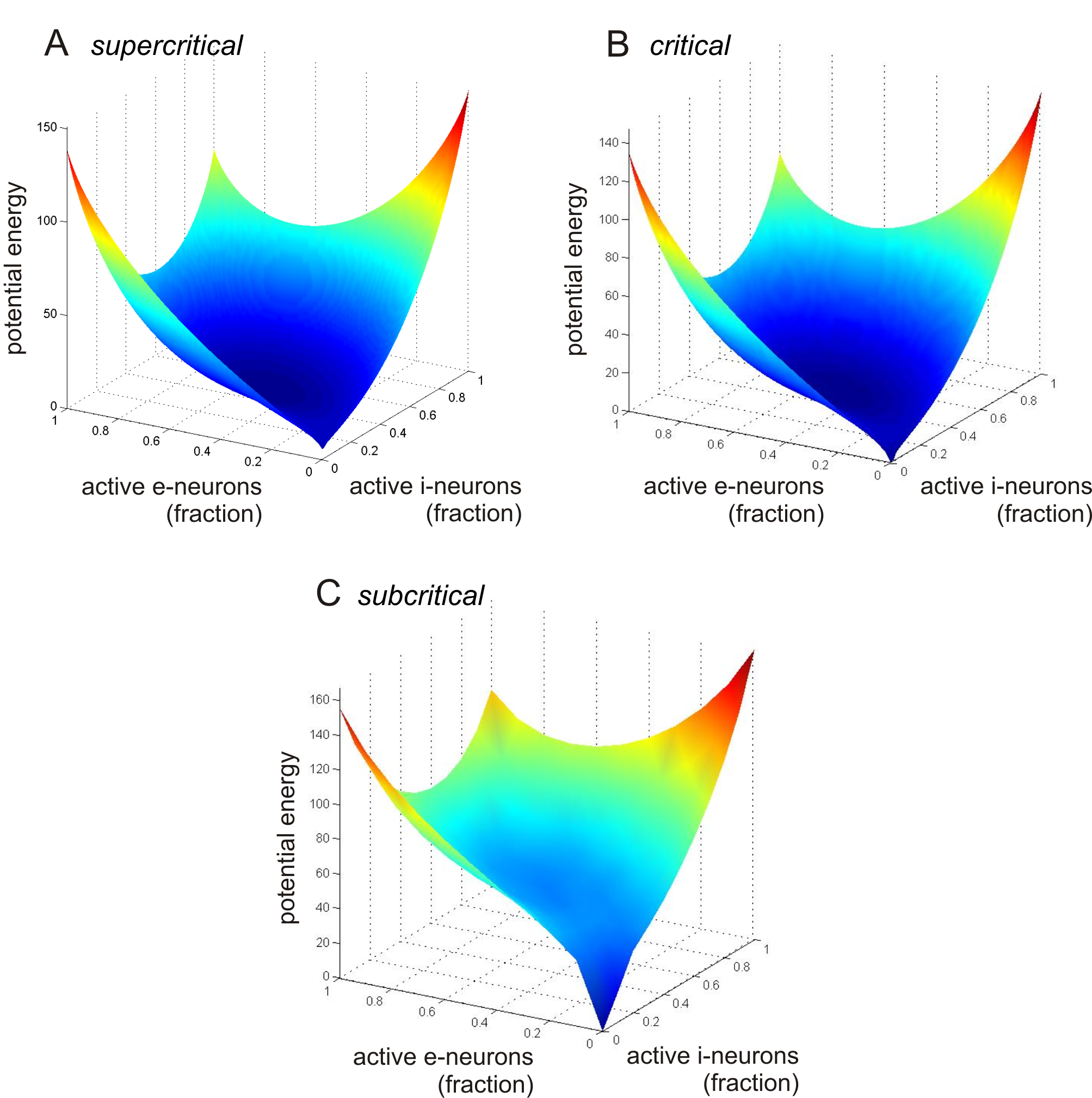

Supplement: Figure S5 — Noise-induced reallocation of the ground state of the stationary potential energy landscape in the NN model. () in (A), () in (B), and () in (C). For network sizes above the critical value , the ground state of the potential energy landscape is at the fixed point , predicted by the macroscopic equations. As the network size decreases from supercritical to subcritical values, the depth of the potential well located at decreases, whereas a new potential well emerges, located at the inactive state , with increasing depth and width. For network sizes below the critical value, noise-induced deformation of the potential energy landscape results in a reallocation of the ground state from to the inactive state . This type of “phase transition” demarcates the onset of avalanching. (TIF) [file pcbi.1003411.s005.tif]

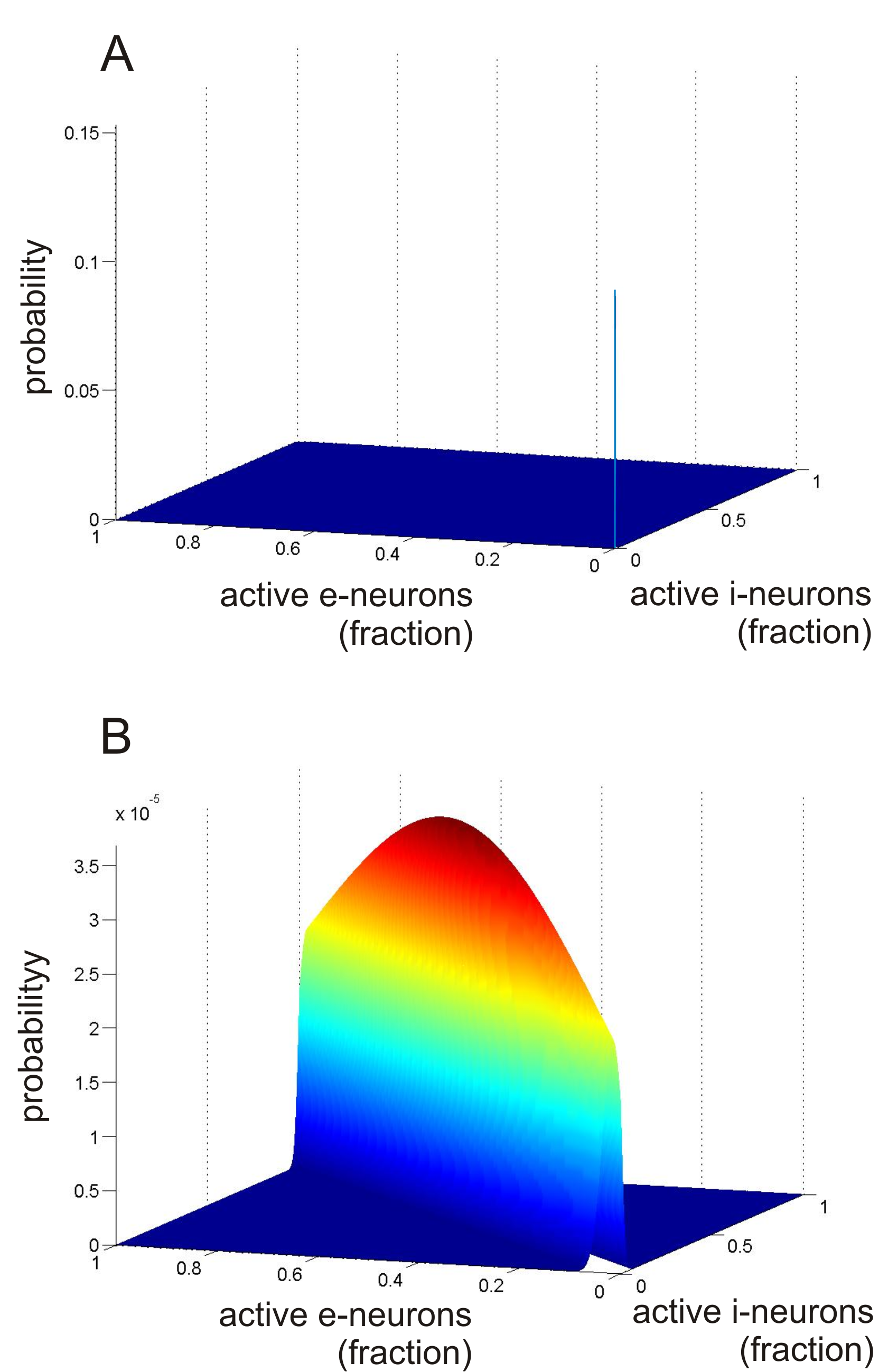

Supplement: Figure S6 — Failure of LNA to sufficiently approximate the stationary probability distribution in the NN model. (A) The true stationary probability of the fractional activity process in the NN model considered in [10], with , , , , and . (B) The approximating stationary probability distribution obtained by the LNA method. Clearly, the LNA method provides a poor approximation to the actual probability distribution in this case. In particular, the true distribution depicted in (A) predicts a probability of 0.45 for the network to be at a state close to the inactive state and a probability of for the network to be at a state within a small neighborhood around the mode , predicted by the macroscopic equations. On the other hand, the corresponding probabilities predicted by the sampled Gaussian distribution depicted in (B) are and . (TIF) [file pcbi.1003411.s006.tif]
