# Supplementary material for: Intrinsic Noise Induces Critical Behavior in Leaky Markovian Networks Leading to Avalanching
Source: PLoS Comput Biol. 2014 Jan 9;10(1):e1003411. doi: 10.1371/journal.pcbi.1003411 (PMC3886886; doi:10.1371/journal.pcbi.1003411)
Supplement: Text S1 — Additional mathematical and modeling details: LMNs and Boolean networks, Markovianity of the fractional activity process, the linear noise approximation method applied to the LMN model, leakiness and irreducibility, definition of thermodynamic quantities and connections to thermodynamic stability, robustness and critical behavior, equilibrium and non-equilibrium behavior, relation between noise-induced modes, stochastic transition and bursting, new definition for avalanches, details on the epidemiological and neural network examples. (PDF) [file pcbi.1003411.s007.pdf]

Intrinsic Noise Induces Critical Behavior in Leaky Markovian Networks Leading to Avalanching

Garrett Jenkinson, **John Goutsias***
Whitaker Biomedical Engineering Institute, The Johns Hopkins University, Baltimore, Maryland, United States of America
$*$ Corresponding author: goutsias@jhu.edu

Supporting Information

LMNs and Boolean Networks

Boolean networks were introduced by S. Kauffman more than 40 years ago as models of gene regulation [1, 2]. Since then, they have been extensively used as simple models for the dynamics of complex networks. A Boolean network is typically formulated as a directed graph with N nodes in a set $\mathcal{N} = \{1, 2, \ldots, N\}$, whose state of the n-th node is characterized by a (deterministic) binary variable $x_n(t)$ taking 0-1 values. The status of each node is influenced by an input function $b_n(\boldsymbol{x})$, which is a Boolean function over a subset of the binary state variables $x_1, x_2, \ldots, x_N$. Given a fixed time step Δt, the state of the Boolean network at time $(m+1)\Delta t$ is determined by *synchronously* updating the states of all nodes in the network at time $m\Delta t$ using the deterministic rule

$$x_n((m+1)\Delta t) = b_n(\boldsymbol{x}(m\Delta t)), \quad \text{for every } n \in \mathcal{N}, \tag{S1}$$

or more general probabilistic updating rules [3, 4].

The vast majority of complex networks of interest do not update their states in a synchronous manner. As a consequence, Boolean networks tend to oversimplify the dynamics of many real networks. To address this problem, a number of investigators have focused their effort on an *asynchronous* stochastic updating scheme that leads to stochastic asynchronous Boolean networks [5–7]. According to this scheme, the state of a Boolean network at time $(m+1)\Delta t$ is determined by randomly selecting a node in the network (usually uniformly among all nodes), by updating the state of this node using the associated Boolean function, and by leaving the states of the remaining nodes unchanged. In this case,

$$x_n((m+1)\Delta t) = \begin{cases} b_{n^*}(\boldsymbol{x}(m\Delta t)), & \text{for } n = n^* \\ x_n(m\Delta t), & \text{for } n \neq n^*, \end{cases} \tag{S2}$$

where n^* is the node selected to be updated at time $(m+1)\Delta t$.

Although the previous modification results in a model than may be more realistic than the classical Boolean model, it does not take into consideration major features of real complex networks. In particular, the model does not account for the facts that state updating can occur at any time t (not necessarily at discrete times $m\Delta t$) and that the time of next updating as well as the node to be updated can be influenced by the current state of the network. To address these issues, a number of models have been proposed in the literature [8–14]. It turns out that the LMN model discussed in this paper effectively addresses these problems and provides a natural alternative to the stochastic Boolean network models studied in the literature; see [14]. As a matter of fact, if the network is at state $\boldsymbol{x}(t)$ at time t, then the time $t + \tau^*$ at which the state of the network will be next updated can be determined by drawing a sample τ^* from the exponential distribution

$$e_t(\tau) = \Big\{ \sum_{n \in \mathcal{N}} \alpha_n(\boldsymbol{x}(t)) \Big\} \exp \Big\{ - \tau \sum_{n \in \mathcal{N}} \alpha_n(\boldsymbol{x}(t)) \Big\}, \; \tau > 0, \tag{S3}$$

where

$$\alpha_n(\boldsymbol{x}) = (1 - x_n)\big[\ell_n^+ + f_n(r_n(\boldsymbol{x}))\big] + x_n\big[\ell_n^- + g_n(r_n(\boldsymbol{x}))\big]. \tag{S4}$$

Moreover, the node to be updated can be specified by drawing a sample n^* from the probability distribution

$$u_t(n) = \frac{\alpha_n(\boldsymbol{x}(t))}{\sum_{n' \in \mathcal{N}} \alpha_{n'}(\boldsymbol{x}(t))}, \; n \in \mathcal{N}. \tag{S5}$$

In this case [compare with Eq. (S2)]

$$x_n(t+\tau^*) = \begin{cases} 1 - x_{n^*}(t), & \text{for } n = n^* \\ x_n(t), & \text{for } n \neq n^*. \end{cases} \tag{S6}$$

This implies that the LMN model is a continuous-time Boolean network model with state-dependent asynchronous node updating and Boolean functions $b_n(\boldsymbol{x}) = 1 - x_n$ assigned at each node $n \in \mathcal{N}$.

Markovianity of the Fractional Activity Process

Because the activity process $\{\boldsymbol{X}(t), t \geq 0\}$ is a Markov process, only a single transition from $\boldsymbol{X}(t)$ to $\boldsymbol{X}(t) + \boldsymbol{e}_{n^*}$ or $\boldsymbol{X}(t) - \boldsymbol{e}_{n^*}$, for some $n^* \in \mathcal{N}$, can occur within the infinitesimally small time interval $[t, t+dt)$, where $\boldsymbol{e}_{n^*}$ is the n^*-th column of the $N \times N$ identity matrix. Since

$$Y_k(t) := \frac{1}{N_k} \sum_{n \in \mathcal{N}_k} X_n(t), \quad \text{for every } k \in \mathcal{K}, \tag{S7}$$

where $\mathcal{K} = \{1, 2, \ldots, K\}$, with K being the number of homogeneous sub-populations $\mathcal{N}_k$, and $N_k = |\mathcal{N}_k|$ (i.e., the cardinality of $\mathcal{N}_k$), this also means that the fractional activity process $\{\boldsymbol{Y}(t), t \geq 0\}$ can transition only once within $[t, t+dt)$ from $\boldsymbol{Y}(t)$ to $\boldsymbol{Y}(t) + \widetilde{\boldsymbol{e}}_k$ or $\boldsymbol{Y}(t) - \widetilde{\boldsymbol{e}}_k$. Here, $\widetilde{\boldsymbol{e}}_k$ is the k-th column of the $K \times K$ identity matrix multiplied by N_k^{-1} and $k \in \mathcal{K}$ is such that $n^* \in \mathcal{N}_k$. As a matter of fact, the transition probabilities are given by

$$\begin{aligned}
\Pr\left[\boldsymbol{Y}(t) \to \boldsymbol{Y}(t) + \widetilde{\boldsymbol{e}}_k \text{ within } [t, t+dt) \mid \boldsymbol{X}(t) = \boldsymbol{x}\right] &= \sum_{n \in \mathcal{N}_k} p_n^+(\boldsymbol{x}) dt \\
&= \sum_{n \in \mathcal{N}_k} (1 - x_n)\left[\ell_n^+ + f_n(r_n(\boldsymbol{x}))\right] dt \\
&= \sum_{n \in \mathcal{N}_k} (1 - x_n)\left[\lambda_k^+ + \phi_k(\rho_k(\boldsymbol{y}))\right] dt \\
&= \Big(N_k - \sum_{n \in \mathcal{N}_k} x_n\Big)\left[\lambda_k^+ + \phi_k(\rho_k(\boldsymbol{y}))\right] dt \\
&= N_k(1 - y_k)\left[\lambda_k^+ + \phi_k(\rho_k(\boldsymbol{y}))\right] dt,
\end{aligned} \tag{S8}$$

where $p_n^+(\boldsymbol{x})$ is the propensity function of activation of the n-th node of the LMN (see Methods in Main Text) and the k-th element y_k of $\boldsymbol{y}$ is given by $N_k^{-1} \sum_{n \in \mathcal{N}_k} x_n$. Moreover, ϕ_k and λ_k^+ are such that, for every $k \in \mathcal{K}$, $f_n = \phi_k$ and $\ell_n^+ = \lambda_k^+$, for all $n \in \mathcal{N}_k$, and we have ignored terms that go to zero faster than dt. Finally, we have used the assumption that there exists a function ρ_k such that $r_n(\boldsymbol{x}) = \rho_k(\boldsymbol{y})$,

for every $n \in \mathcal{N}_k$. Likewise, we have that

$$
\begin{aligned}
\Pr\big[\boldsymbol{Y}(t) \to \boldsymbol{Y}(t) - \widetilde{\boldsymbol{e}}_k \text{ within } [t, t+dt) \mid \boldsymbol{X}(t) = \boldsymbol{x}\big] &= \sum_{n \in \mathcal{N}_k} p_n^-(\boldsymbol{x}) dt \\
&= \sum_{n \in \mathcal{N}_k} x_n \big[\ell_n^- + g_n(r_n(\boldsymbol{x}))\big] dt \\
&= \sum_{n \in \mathcal{N}_k} x_n \big[\lambda_k^- + \gamma_k(\rho_k(\boldsymbol{y}))\big] dt \\
&= N_k y_k \big[\lambda_k^- + \gamma_k(\rho_k(\boldsymbol{y}))\big] dt, \qquad \text{(S9)}
\end{aligned}
$$

where $p_n^-(\boldsymbol{x})$ is the propensity function of inactivation of the n-th node of the LMN (see Methods in Main Text), whereas γ_k and λ_k^- are such that, for every $k \in \mathcal{K}$, $g_n = \gamma_k$ and $\ell_n^- = \lambda_k^-$, for all $n \in \mathcal{N}_k$.

The previous discussion shows that the fractional activity process $\{\boldsymbol{Y}(t), t \geq 0\}$ is Markovian with propensity functions given by

$$\pi_k^+(\boldsymbol{y}) = N_k(1 - y_k)\big[\lambda_k^+ + \phi_k(\rho_k(\boldsymbol{y}))\big] \qquad \text{(S10)}$$

$$\pi_k^-(\boldsymbol{y}) = N_k y_k \big[\lambda_k^- + \gamma_k(\rho_k(\boldsymbol{y}))\big]. \qquad \text{(S11)}$$

As a consequence, the probability distribution $P(\boldsymbol{y}; t)$ satisfies the following master equation:

$$\frac{\partial P(\boldsymbol{y}; t)}{\partial t} = \sum_{k \in \mathcal{K}} \Big\{ \pi_k^+(\boldsymbol{y} - \widetilde{\boldsymbol{e}}_k) P(\boldsymbol{y} - \widetilde{\boldsymbol{e}}_k; t) + \pi_k^-(\boldsymbol{y} + \widetilde{\boldsymbol{e}}_k) P(\boldsymbol{y} + \widetilde{\boldsymbol{e}}_k; t) - \big[\pi_k^+(\boldsymbol{y}) + \pi_k^-(\boldsymbol{y})\big] P(\boldsymbol{y}; t) \Big\}. \qquad \text{(S12)}$$

Finally, it is important to note that, when the net input to a node n in the LMN is given by $r_n(\boldsymbol{x}) = h_n + \boldsymbol{a}_n^T \boldsymbol{x}$, we can find a function ρ_k such that $r_n(\boldsymbol{x}) = \rho_k(\boldsymbol{y})$, for every $n \in \mathcal{N}_k$. Indeed, if $h_n = \eta_k$, for every $n \in \mathcal{N}_k$, and $a_{nn'} = w_{kk'}/N_{k'}$, for every $n \in \mathcal{N}_k$, $n' \in \mathcal{N}_{k'}$, then, for every $n \in \mathcal{N}_k$, we have that

$$
\begin{aligned}
r_n(\boldsymbol{x}) &= h_n + \sum_{n' \in \mathcal{N}} a_{nn'} x_{n'} \\
&= h_n + \sum_{k' \in \mathcal{K}} \sum_{n' \in \mathcal{N}_{k'}} a_{nn'} x_{n'} \\
&= \eta_k + \sum_{k' \in \mathcal{K}} \sum_{n' \in \mathcal{N}_{k'}} \frac{w_{kk'}}{N_{k'}} x_{n'} \\
&= \eta_k + \sum_{k' \in \mathcal{K}} w_{kk'} \frac{1}{N_{k'}} \sum_{n' \in \mathcal{N}_{k'}} x_{n'} \\
&= \eta_k + \sum_{k' \in \mathcal{K}} w_{kk'} y_{k'}, \qquad \text{(S13)}
\end{aligned}
$$

which shows that $\rho_k(\boldsymbol{y}) = \eta_k + \sum_{k' \in \mathcal{K}} w_{kk'} y_{k'}$.

Linear Noise Approximation

By following [15] (see also [16]), we define the shift operators Σ_k^- and Σ_k^+ as

$$\Sigma_k^- \varphi(y_1, \ldots, y_{k-1}, y_k, y_{k+1}, \ldots, y_K) := \varphi(y_1, \ldots, y_{k-1}, y_k - N_k^{-1}, y_{k+1}, \ldots, y_K) \qquad \text{(S14)}$$

$$\Sigma_k^+ \varphi(y_1, \ldots, y_{k-1}, y_k, y_{k+1}, \ldots, y_K) := \varphi(y_1, \ldots, y_{k-1}, y_k + N_k^{-1}, y_{k+1}, \ldots, y_K), \qquad \text{(S15)}$$

for $k \in \mathcal{K}$ and any function $\varphi(\boldsymbol{y})$. By using a Taylor series expansion, we have that

$$\Sigma_k^- \varphi(\boldsymbol{y}) = \varphi(\boldsymbol{y}) - N_k^{-1} \frac{\partial \varphi(\boldsymbol{y})}{\partial y_k} + \frac{N_k^{-2}}{2} \frac{\partial^2 \varphi(\boldsymbol{y})}{\partial y_k^2} - \frac{N_k^{-3}}{3!} \frac{\partial^3 \varphi(\boldsymbol{y})}{\partial y_k^3} + \cdots \tag{S16}$$

$$\Sigma_k^+ \varphi(\boldsymbol{y}) = \varphi(\boldsymbol{y}) + N_k^{-1} \frac{\partial \varphi(\boldsymbol{y})}{\partial y_k} + \frac{N_k^{-2}}{2} \frac{\partial^2 \varphi(\boldsymbol{y})}{\partial y_k^2} + \frac{N_k^{-3}}{3!} \frac{\partial^3 \varphi(\boldsymbol{y})}{\partial y_k^3} + \cdots \tag{S17}$$

In this case, we can write the master equation (S12) as

$$\begin{aligned}
\frac{\partial P(\boldsymbol{y};t)}{\partial t} &= \sum_{k \in \mathcal{K}} \Big\{ \big(\Sigma_k^- - 1\big)\, \pi_k^+(\boldsymbol{y}) P(\boldsymbol{y};t) + \big(\Sigma_k^+ - 1\big)\, \pi_k^-(\boldsymbol{y}) P(\boldsymbol{y};t) \Big\} \\
&= \sum_{k \in \mathcal{K}} \Big\{ \Big(- N_k^{-1} \frac{\partial}{\partial y_k} + \frac{N_k^{-2}}{2} \frac{\partial^2}{\partial y_k^2} - \frac{N_k^{-3}}{3!} \frac{\partial^3}{\partial y_k^3} + \cdots \Big) \times \pi_k^+(\boldsymbol{y}) P(\boldsymbol{y};t) \\
&\qquad + \Big(N_k^{-1} \frac{\partial}{\partial y_k} + \frac{N_k^{-2}}{2} \frac{\partial^2}{\partial y_k^2} + \frac{N_k^{-3}}{3!} \frac{\partial^3}{\partial y_k^3} + \cdots \Big) \times \pi_k^-(\boldsymbol{y}) P(\boldsymbol{y};t) \Big\}.
\end{aligned} \tag{S18}$$

If we now define the drift and diffusion functions by

$$A_k(\boldsymbol{y}) := \frac{\pi_k^+(\boldsymbol{y}) - \pi_k^-(\boldsymbol{y})}{N_k} \tag{S19}$$

$$D_k(\boldsymbol{y}) := \frac{\pi_k^+(\boldsymbol{y}) + \pi_k^-(\boldsymbol{y})}{N_k}, \tag{S20}$$

respectively, then

$$\frac{\partial P(\boldsymbol{y};t)}{\partial t} = - \sum_{k \in \mathcal{K}} \Big\{ \frac{\partial [A_k(\boldsymbol{y}) P(\boldsymbol{y};t)]}{\partial y_k} - \frac{N_k^{-1}}{2} \frac{\partial^2 [D_k(\boldsymbol{y}) P(\boldsymbol{y};t)]}{\partial y_k^2} + \frac{N_k^{-2}}{3!} \frac{\partial^3 [A_k(\boldsymbol{y}) P(\boldsymbol{y};t)]}{\partial y_k^3} + \cdots \Big\}. \tag{S21}$$

This equation, together with the *ansatz*

$$Y_k(t) = \mu_k(t) + \frac{1}{\sqrt{N_k}}\, W_k(t), \quad t > 0, \quad k \in \mathcal{K}, \tag{S22}$$

yields

$$\frac{\partial P(\boldsymbol{y};t)}{\partial t} = - \sum_{k \in \mathcal{K}} \Big\{ N_k^{1/2} \frac{\partial [A_k(\boldsymbol{y}) P(\boldsymbol{y};t)]}{\partial w_k} - \frac{1}{2} \frac{\partial^2 [D_k(\boldsymbol{y}) P(\boldsymbol{y};t)]}{\partial w_k^2} + \frac{N_k^{-1/2}}{3!} \frac{\partial^3 [A_k(\boldsymbol{y}) P(\boldsymbol{y};t)]}{\partial w_k^3} + \cdots \Big\}. \tag{S23}$$

In Eq. (S22), $\boldsymbol{\mu}(t)$ solves the macroscopic differential equations:

$$\frac{d\mu_k(t)}{dt} = [1 - \mu_k(t)]\big[\lambda_k^+ + \phi_k(\rho_k(\boldsymbol{\mu}(t)))\big] - \mu_k(t)\big[\lambda_k^- + \gamma_k(\rho_k(\boldsymbol{\mu}(t)))\big], \; t > 0, \; k \in \mathcal{K}, \tag{S24}$$

initialized by $\boldsymbol{\mu}(0) = \mathbf{0}$, whereas, for each t, $W_k(t)$, $k \in \mathcal{K}$, are zero-mean correlated Gaussian random variables. We can now use a Taylor expansion of the drift and diffusion functions to obtain:

$$\begin{aligned}
A_k(\boldsymbol{y}) &= A_k\Big(\mu_1 + \frac{1}{\sqrt{N_1}}\, w_1, \ldots, \mu_K + \frac{1}{\sqrt{N_K}}\, w_K\Big) \\
&= A_k(\boldsymbol{\mu}) + \sum_{k' \in \mathcal{K}} N_{k'}^{-1/2} w_{k'} A_{kk'}(\boldsymbol{\mu}) + \sum_{k' \in \mathcal{K}} \sum_{k'' \in \mathcal{K}} N_{k'}^{-1/2} N_{k''}^{-1/2} w_{k'} w_{k''} A_{kk'k''}(\boldsymbol{\mu}) + \cdots ,
\end{aligned} \tag{S25}$$

for $k \in \mathcal{K}$, where $A_{kk'}(\boldsymbol{y}) := \partial A_k(\boldsymbol{y})/\partial y_{k'}$ and $A_{kk'k''}(\boldsymbol{y}) := \partial^2 A_k(\boldsymbol{y})/\partial y_{k'}\partial y_{k''}$, and likewise for the diffusion functions. In this case, Eq. (S23) becomes

$$\frac{\partial P(\boldsymbol{y};t)}{\partial t} = -\sum_{k\in\mathcal{K}} N_k^{1/2} \frac{\partial[A_k(\boldsymbol{\mu})P(\boldsymbol{y};t)]}{\partial w_k} - \sum_{k\in\mathcal{K}} N_k^{1/2} \sum_{k'\in\mathcal{K}} N_{k'}^{-1/2} \frac{\partial[w_{k'}A_{kk'}(\boldsymbol{\mu})P(\boldsymbol{y};t)]}{\partial w_k}$$
$$+\frac{1}{2}\sum_{k\in\mathcal{K}} \frac{\partial^2[D_k(\boldsymbol{\mu})P(\boldsymbol{y};t)]}{\partial w_k^2} + \mathcal{O}(N_k^{-1/2}). \tag{S26}$$

We can now replace the probability distribution $P(\boldsymbol{y};t)$ of the fractional activity process with the probability distribution $P'(\boldsymbol{w};t)$ of the noise process, in which case we obtain

$$\frac{\partial P'(\boldsymbol{w};t)}{\partial t} = \sum_{k\in\mathcal{K}} N_k^{1/2}\left[\frac{d\mu_k(t)}{dt} - A_k(\boldsymbol{\mu}(t))\right]\frac{\partial P'(\boldsymbol{w};t)}{\partial w_k}$$
$$-\sum_{k\in\mathcal{K}} N_k^{1/2}\sum_{k'\in\mathcal{K}} N_{k'}^{-1/2} A_{kk'}(\boldsymbol{\mu}(t))\,\frac{\partial[w_{k'}P'(\boldsymbol{w};t)]}{\partial w_k}$$
$$+\frac{1}{2}\sum_{k\in\mathcal{K}} D_k(\boldsymbol{\mu}(t))\,\frac{\partial^2 P'(\boldsymbol{w};t)}{\partial w_k^2} + \mathcal{O}(N_k^{-1/2}), \tag{S27}$$

by virtue of the fact that

$$\frac{\partial P(\boldsymbol{y};t)}{\partial t} = \frac{\partial P'(\boldsymbol{w};t)}{\partial t} + \sum_{k\in\mathcal{K}} \frac{dw_k(t)}{dt}\frac{\partial P'(\boldsymbol{w};t)}{\partial w_k}$$
$$= \frac{\partial P'(\boldsymbol{w};t)}{\partial t} - \sum_{k\in\mathcal{K}} N_k^{1/2}\frac{d\mu_k(t)}{dt}\frac{\partial P'(\boldsymbol{w};t)}{\partial w_k}, \tag{S28}$$

where the second equality is a consequence of the fact that $dY_k(t)/dt = 0$ (except at time points on a set of measure zero at which the derivative is infinite). This implies that $dw_k(t)/dt = -N_k^{1/2} d\mu_k(t)/dt$, by virtue of Eq. (S22).

Note now that $\boldsymbol{\mu}(t)$ satisfies the macroscopic equations $d\mu_k(t)/dt = A_k(\boldsymbol{\mu}(t))$. As a consequence, and by virtue of Eq. (S27), the noise probability distribution $P'(\boldsymbol{w};t)$ is approximately governed by the *linear* Fokker-Planck equation

$$\frac{\partial P'(\boldsymbol{w};t)}{\partial t} = -\sum_{k\in\mathcal{K}}\sum_{k'\in\mathcal{K}}\sqrt{\frac{\zeta_k}{\zeta_{k'}}}\,A_{kk'}(\boldsymbol{\mu}(t))\,\frac{\partial[w_{k'}P'(\boldsymbol{w};t)]}{\partial w_k} + \frac{1}{2}\sum_{k\in\mathcal{K}} D_k(\boldsymbol{\mu}(t))\,\frac{\partial^2 P'(\boldsymbol{w};t)}{\partial w_k^2}, \tag{S29}$$

where $\zeta_k = N_k/N$ and we ignore the terms of $\mathcal{O}(N_k^{-1/2})$. Note that this equation does not depend on N, since we have used the relation $N_k/N_{k'} = \zeta_k/\zeta_{k'}$, which is true at any point *en route* to the thermodynamic limit. The solution to this equation is a multivariate Gaussian density with zero mean and correlation matrix $\mathbb{R}(t)$ with elements $r_{kk'}(t) = \mathrm{E}[W_k(t)W_{k'}(t)]$ that satisfy the following system of Lyapunov equations:

$$\frac{dr_{kk'}(t)}{dt} = D_k(\boldsymbol{\mu}(t))\Delta(k-k') + \sum_{k''\in\mathcal{K}}\sqrt{\frac{\zeta_k}{\zeta_{k''}}}\,A_{kk''}(\boldsymbol{\mu}(t))\,r_{k''k'}(t) + \sum_{k''\in\mathcal{K}}\sqrt{\frac{\zeta_{k'}}{\zeta_{k''}}}\,A_{k'k''}(\boldsymbol{\mu}(t))\,r_{kk''}(t), \tag{S30}$$

for $t > 0$ and $k, k' \in \mathcal{K}$, initialized with $r_{kk'}(0) = 0$, for every $k, k' \in \mathcal{K}$, where Δ is the Kronecker delta function.

We note here that there are two approximation steps involved with the LNA method. The first step is the *ansatz* given by Eq. (S22), whereas the second step is ignoring all terms of $\mathcal{O}(N_k^{-1/2})$ in Eq. (S27). Theoretically speaking, justification of the second step is simple – we can take N to be large so that $1/\sqrt{N_k}$ is small enough that any term in the expansion multiplied by $1/\sqrt{N_k}$ is negligible. This requirement however is not useful in practice, since we cannot determine the appropriate value of N that satisfies the required condition. On the other hand, assuming that N is large enough for the previous approximation to be valid, Eq. (S22) can be justified only for *monostable* systems [15].

Leakiness and Irreducibility

In the theory of Markov processes, the state $\boldsymbol{y}$ is said to be *accessible* from another state $\boldsymbol{y}'$ if there is a non-zero probability to transition (possibly through intermediate states) from $\boldsymbol{y}'$ to $\boldsymbol{y}$. We denote this by $\boldsymbol{y}' \to \boldsymbol{y}$. The states $\boldsymbol{y}$ and $\boldsymbol{y}'$ are said to be *communicating* whenever $\boldsymbol{y} \to \boldsymbol{y}'$ and $\boldsymbol{y}' \to \boldsymbol{y}$. In this case, we write $\boldsymbol{y} \rightleftarrows \boldsymbol{y}'$. If all states $\boldsymbol{y} \in \mathcal{Y}$ are communicating, then there is a non-zero probability to transition from any state to any other state, and the Markov process is said to be *irreducible*. An irreducible Markov process has a unique, strictly positive, and asymptotically stable stationary probability distribution that is independent of the initial condition.

It is often difficult to prove that a Markov processes is irreducible. The following results allow us to determine when the fractional activity process $\boldsymbol{Y}(t)$ in a LMN is irreducible. It turns out that leakiness is intimately related to irreducibility.

Proposition 1. *If $\lambda_k^+ > 0$, for every $k \in \mathcal{K}$, then $\mathbf{0} \to \boldsymbol{y}' \to \mathbf{1}$ for all $\boldsymbol{y}' \in \mathcal{Y}$, where $\mathbf{0}$ is the state of zero fractional inactivity and $\mathbf{1}$ is the state of maximum fractional activity.*

Proof. For every $\boldsymbol{y} \in \mathcal{Y}$ such that $y_k \leq 1 - 1/N_k$, for some $k \in \mathcal{K}$, we have $\pi_k^+(\boldsymbol{y}) = N_k(1-y_k)[\lambda_k^+ + \phi_k(\rho_k(\boldsymbol{y}))] \geq \lambda_k^+ + \phi_k(\rho_k(\boldsymbol{y})) > 0$, where the second inequality is due to the fact that $\lambda_k^+ > 0$ and $\phi_k(\rho_k(\boldsymbol{y})) \geq 0$. Therefore, $\boldsymbol{y} \to \boldsymbol{y} + \widetilde{\boldsymbol{e}}_k$, where $\widetilde{\boldsymbol{e}}_k$ is the k-th column of the $K \times K$ identity matrix multiplied by N_k^{-1}. Thus, for any state $\boldsymbol{y} \in \mathcal{Y}$ such that $\boldsymbol{y} + \widetilde{\boldsymbol{e}}_k \in \mathcal{Y}$, we have that $\boldsymbol{y} \to \boldsymbol{y} + \widetilde{\boldsymbol{e}}_k$. Let $\boldsymbol{y}' = (n_1/N_1, n_2/N_2, \ldots, n_K/N_K)^T$, for some $n_1, n_2, \ldots, n_K$. A non-zero probability path from $\mathbf{0}$ to $\boldsymbol{y}'$ is then given by the following sequence: n_1 transitions taking $\boldsymbol{y} \to \boldsymbol{y} + \widetilde{\boldsymbol{e}}_1$, followed by n_2 transitions taking $\boldsymbol{y} \to \boldsymbol{y} + \widetilde{\boldsymbol{e}}_2, \ldots$, followed by n_K transitions taking $\boldsymbol{y} \to \boldsymbol{y} + \widetilde{\boldsymbol{e}}_K$. Likewise, a non-zero probability path from $\boldsymbol{y}'$ to $\mathbf{1}$ is given by the following sequence: $N_1 - n_1$ transitions taking $\boldsymbol{y} \to \boldsymbol{y} + \widetilde{\boldsymbol{e}}_1$, followed by $N_2 - n_2$ transitions taking $\boldsymbol{y} \to \boldsymbol{y} + \tilde{\boldsymbol{e}}_2, \ldots$, followed by $N_K - n_K$ transitions taking $\boldsymbol{y} \to \boldsymbol{y} + \widetilde{\boldsymbol{e}}_K$. Hence, $\mathbf{0} \to \boldsymbol{y}' \to \mathbf{1}$, for all $\boldsymbol{y}' \in \mathcal{Y}$. □

Corollary 1. *If $\lambda_k^+ > 0$, for every $k \in \mathcal{K}$, and $\mathbf{1} \to \mathbf{0}$, then $\boldsymbol{Y}(t)$ is irreducible.*

Proof. From Proposition 1, and for any $\boldsymbol{y}', \boldsymbol{y}'' \in \mathcal{Y}$, we have that $\boldsymbol{y}' \to \mathbf{1}$ and $\mathbf{0} \to \boldsymbol{y}''$. Since, $\mathbf{1} \to \mathbf{0}$, this implies $\boldsymbol{y}' \to \mathbf{1} \to \mathbf{0} \to \boldsymbol{y}''$ and thus $\boldsymbol{Y}(t)$ is irreducible. □

Proposition 2. *If $\lambda_k^- > 0$, for every $k \in \mathcal{K}$, then $\mathbf{1} \to \boldsymbol{y}' \to \mathbf{0}$ for all $\boldsymbol{y}' \in \mathcal{Y}$.*

Proof. For every $\boldsymbol{y} \in \mathcal{Y}$ such that $y_k \geq 1/N_k$, for some $k \in \mathcal{K}$, we have $\pi_k^-(\boldsymbol{y}) = N_k y_k[\lambda_k^- + \gamma_k(\rho_k(\boldsymbol{y}))] \geq \lambda_k^- + \gamma_k(\rho_k(\boldsymbol{y})) > 0$, where the second inequality is due to the fact that $\lambda_k^- > 0$ and $\gamma_k(\rho_k(\boldsymbol{y})) \geq 0$. Therefore, $\boldsymbol{y} \to \boldsymbol{y} - \widetilde{\boldsymbol{e}}_k$. Thus, for any state $\boldsymbol{y} \in \mathcal{Y}$ such that $\boldsymbol{y} - \widetilde{\boldsymbol{e}}_k \in \mathcal{Y}$, we have that $\boldsymbol{y} \to \boldsymbol{y} - \widetilde{\boldsymbol{e}}_k$. Let $\boldsymbol{y}' = (n_1/N_1, n_2/N_2, \ldots, n_K/N_K)^T$, for some $n_1, n_2, \ldots, n_K$. A non-zero probability path from $\mathbf{1}$ to $\boldsymbol{y}'$ is then given by the following sequence: $N_1 - n_1$ transitions taking $\boldsymbol{y} \to \boldsymbol{y} - \widetilde{\boldsymbol{e}}_1$, followed by $N_2 - n_2$ transitions taking $\boldsymbol{y} \to \boldsymbol{y} - \widetilde{\boldsymbol{e}}_2, \ldots$, followed by $N_K - n_K$ transitions taking $\boldsymbol{y} \to \boldsymbol{y} - \widetilde{\boldsymbol{e}}_K$. On the other hand, a non-zero probability path from $\boldsymbol{y}'$ to $\mathbf{0}$ is given by the following sequence: n_1 transitions taking $\boldsymbol{y} \to \boldsymbol{y} - \widetilde{\boldsymbol{e}}_1$, followed by n_2 transitions taking $\boldsymbol{y} \to \boldsymbol{y} - \widetilde{\boldsymbol{e}}_2, \ldots$, followed by n_K transitions taking $\boldsymbol{y} \to \boldsymbol{y} - \widetilde{\boldsymbol{e}}_K$. Hence, $\mathbf{1} \to \boldsymbol{y}' \to \mathbf{0}$, for all $\boldsymbol{y}' \in \mathcal{Y}$. □

Corollary 2. *If* $\lambda_k^- > 0$*, for every* $k \in \mathcal{K}$*, and* $\mathbf{0} \to \mathbf{1}$*, then* $\boldsymbol{Y}(t)$ *is irreducible.*

Proof. From Proposition 2, and for any $\boldsymbol{y}', \boldsymbol{y}'' \in \mathcal{Y}$, we have that $\boldsymbol{y}' \to \mathbf{0}$ and $\mathbf{1} \to \boldsymbol{y}''$. Since, $\mathbf{0} \to \mathbf{1}$, this implies $\boldsymbol{y}' \to \mathbf{0} \to \mathbf{1} \to \boldsymbol{y}''$ and thus $\boldsymbol{Y}(t)$ is irreducible. □

By combining the previous results, we obtain the following theorem:

Theorem 1. *If* $\lambda_k^-, \lambda_k^+ > 0$*, for every* $k \in \mathcal{K}$*, then* $\boldsymbol{Y}(t)$ *is irreducible.*

Note that physical systems satisfy a condition referred to as *microscopic reversibility.* This means that any physically possible transition from one state to another is also possible in the reverse direction. According to Theorem 1, taking $\lambda_k^-, \lambda_k^+ > 0$, for every $k \in \mathcal{K}$, results in an irreducible LMN model that ensures microscopic reversibility since, in this case, transitions from one state to another are also possible in the reverse direction. Therefore, we can make a LMN that violates microscopic reversibility satisfy this condition by setting the leak parameters to positive (and, if necessary, minuscule) values.

Thermodynamic Stability, Robustness, and Critical Behavior

We now employ a parameter $\Omega = N/N_0$, where N is the net number of nodes in the network and N_0 is a normalizing constant. If $N_0 \gg 1$, we may approximately assume that Ω is continuous-valued. We use Ω to quantify the size of the network.

The probability distribution of the fractional activity process is given by the Boltzmann-Gibbs distribution

$$P_\Omega(\boldsymbol{y}; t) = \frac{1}{Z_\Omega(t)}\, e^{-\Omega V_\Omega(\boldsymbol{y}; t)}, \tag{S31}$$

where

$$V_\Omega(\boldsymbol{y}; t) := -\frac{1}{\Omega} \ln \frac{P_\Omega(\boldsymbol{y}; t)}{P_\Omega(\boldsymbol{y}^*_\Omega(t); t)} \tag{S32}$$

is the potential energy function with $\boldsymbol{y}^*_\Omega(t)$ being a state in $\mathcal{Y}$ at which $P_\Omega(\boldsymbol{y}; t)$ attains its (global) maximum at time t, and

$$Z_\Omega(t) := \sum_{\boldsymbol{y} \in \mathcal{Y}} e^{-\Omega V_\Omega(\boldsymbol{y}; t)} \tag{S33}$$

is the partition function. Based on this formula, we can define a number of quantities that can be used to characterize the behavior of a LMN (and, as a matter of fact, of any Markovian network [17]), when nodes are removed from the network. We adopt these quantities from classical thermodynamics where they are used to describe the behavior of a physical system as its volume contracts or expands [18–21]. In particular, we can define the internal energy, entropy, and Helmholtz free energy, and subsequently introduce the concepts of internal potential energy, free potential energy, internal pressure, pressure, and bulk modulus. In the following, we denote by $\overline{A}$ the stationary limit of a time-varying parameter $A(t)$; i.e., $\overline{A} = \lim_{t \to \infty} A(t)$.

The internal energy at time t is defined by

$$\mathcal{U}_\Omega(t) := \Omega \mathrm{E}_t[U_\Omega(\boldsymbol{Y})], \quad \text{for} \;\; t \geq 0, \tag{S34}$$

where

$$U_\Omega(\boldsymbol{y}) := -\frac{1}{\Omega} \ln \overline{P}_\Omega(\boldsymbol{y}) \tag{S35}$$

is the energy of state $\boldsymbol{y} \in \mathcal{Y}$ and $\mathrm{E}_t[\cdot]$ denotes expectation with respect to the probability distribution $P_\Omega(\boldsymbol{y}; t)$. On the other hand, the Helmholtz free energy $\mathcal{F}_\Omega(t)$ at time t is defined by

$$\mathcal{F}_\Omega(t) := \mathcal{U}_\Omega(t) - \mathcal{S}_\Omega(t), \tag{S36}$$

where $\mathcal{S}_\Omega(t)$ is the entropy at time t, given by

$$\mathcal{S}_\Omega(t) := -\mathrm{E}_t[\ln P_\Omega(\boldsymbol{Y};t)]. \tag{S37}$$

In thermodynamic terms, the Helmholtz free energy measures the energy available in the LMN to do work when the number of nodes is kept fixed. Note that $\mathcal{U}_\Omega(t) \geq 0$ and $0 \leq \mathcal{S}_\Omega(t) \leq \ln|\mathcal{Y}|$, for every Ω and $t \geq 0$, where $|\mathcal{Y}|$ is the cardinality of the state-space $\mathcal{Y}$. It also turns out that $\mathcal{F}_\Omega(t) \geq 0$ and $d\mathcal{F}_\Omega(t)/dt \leq 0$, for every Ω and $t \geq 0$, with equality only at steady-state [17,22,23].

From Eqs. (S32), (S34) & (S35), we can show that

$$\mathcal{U}_\Omega(t) = \mathcal{V}_\Omega(t) + \Omega U_\Omega(\overline{\boldsymbol{y}}^*_\Omega), \tag{S38}$$

where

$$\mathcal{V}_\Omega(t) := \Omega \mathrm{E}_t[\overline{V}_\Omega(\boldsymbol{Y})], \quad \text{for} \;\; t \geq 0, \tag{S39}$$

is the internal potential energy of the LMN. Moreover,

$$\mathcal{F}_\Omega(t) := \mathcal{A}_\Omega(t) + \Omega U_\Omega(\overline{\boldsymbol{y}}^*_\Omega), \tag{S40}$$

by virtue of Eqs. (S36) & (S38), where

$$\mathcal{A}_\Omega(t) := \mathcal{V}_\Omega(t) - \mathcal{S}_\Omega(t). \tag{S41}$$

Note that $\mathcal{A}_\Omega(t)$ is the portion of the Helmholtz free energy due to the internal potential energy of the LMN. For this reason, we refer to $\mathcal{A}_\Omega(t)$ as the free potential energy. In thermodynamic terms, the free potential energy measures the portion of the energy, not accounted for by the energy of the most likely state, available in the LMN to do work when the number of nodes is kept fixed.

The quantities

$$\mathcal{P}^0_\Omega(t) := \frac{\partial \mathcal{V}_\Omega(t)}{\partial \Omega} \tag{S42}$$

$$\mathcal{P}_\Omega(t) := -\frac{\partial \mathcal{A}_\Omega(t)}{\partial \Omega} \tag{S43}$$

$$\mathcal{B}_\Omega(t) := -\Omega \frac{\partial \mathcal{P}_\Omega(t)}{\partial \Omega} \tag{S44}$$

define the internal pressure, pressure, and bulk modulus of the LMN, respectively. The internal pressure quantifies the rate of change in internal potential energy with respect to a change in the number of nodes, whereas the pressure quantifies the rate of change in free potential energy. Moreover, the bulk modulus measures the network's resistance to changing pressure. Note that $\mathcal{P}_\Omega(t) = \mathcal{Q}_\Omega(t) - \mathcal{P}^0_\Omega(t)$, where

$$\mathcal{Q}_\Omega(t) := \frac{\partial \mathcal{S}_\Omega(t)}{\partial \Omega} \tag{S45}$$

is the rate of entropy change with respect to a change in the number of nodes. Moreover, a network with near zero bulk modulus experiences negligible changes in pressure under changes in the number of nodes. The inverse bulk modulus is known as *compressibility.*

In the following, we focus our interest on the stationary behavior of a LMN. Due to the irreducibility properties of LMNs, all stationary thermodynamic quantities are unique and characteristic to the particular network under consideration. From Eqs. (S32) & (S39), note that

$$\begin{aligned} \overline{\mathcal{V}}_\Omega &= \mathrm{E}[-\ln \overline{P}_\Omega(\boldsymbol{Y})] - [-\ln \overline{P}_\Omega(\overline{\boldsymbol{y}}^*_N)] \\ &= \mathrm{E}[\mathcal{I}_\Omega(\boldsymbol{Y})] - \mathcal{I}_\Omega(\overline{\boldsymbol{y}}^*_\Omega) \geq 0, \end{aligned} \tag{S46}$$

where E[·] denotes expectation with respect to the stationary probability distribution $\overline{P}_\Omega(\boldsymbol{y})$ and

$$\mathcal{I}_\Omega(\boldsymbol{y}) := -\ln \overline{P}_\Omega(\boldsymbol{y}) = \Omega U_\Omega(\boldsymbol{y}). \tag{S47}$$

The function $\mathcal{I}_\Omega(\boldsymbol{y})$ quantifies the amount of information associated with the occurrence of state $\boldsymbol{y}$ at steady-state, known as the self-information of state $\boldsymbol{y}$. Therefore, and from an information-theoretic perspective, the internal potential energy measures how far the self-information of the most likely state at steady-state is from the expected self-information of all network states (which is the entropy). Note that zero internal potential energy implies zero self-information for the most likely state. In this case, the network will be at the most likely state with probability one. As a consequence, we may consider the internal potential energy as a thermodynamic measure of the "stability" of a particular ground state of the stationary potential energy landscape $\overline{V}_\Omega(\boldsymbol{y})$ with smaller values indicating increasing stability of that state.

From Eqs. (S31), (S37), (S39), (S41) & (S47), we also have that

$$\begin{aligned}
\overline{\mathcal{A}}_\Omega &= \sum_{\boldsymbol{y}\in\mathcal{Y}} \Omega \overline{V}_\Omega(\boldsymbol{y}) \overline{P}_\Omega(\boldsymbol{y}) + \sum_{\boldsymbol{y}\in\mathcal{Y}} \big[\ln \overline{P}_\Omega(\boldsymbol{y})\big] \overline{P}_\Omega(\boldsymbol{y}) \\
&= \sum_{\boldsymbol{y}\in\mathcal{Y}} \Omega \overline{V}_\Omega(\boldsymbol{y}) \overline{P}_\Omega(\boldsymbol{y}) - \sum_{\boldsymbol{y}\in\mathcal{Y}} \Omega \overline{V}_\Omega(\boldsymbol{y}) \overline{P}_\Omega(\boldsymbol{y}) - \ln \overline{Z}_\Omega \\
&= -\ln \overline{Z}_\Omega \\
&= \ln \overline{P}_\Omega(\overline{\boldsymbol{y}}^*_\Omega) \\
&= -\mathcal{I}_\Omega(\overline{\boldsymbol{y}}^*_\Omega) \leq 0.
\end{aligned} \tag{S48}$$

Therefore, the negative of the free potential energy is the self-information of the most likely state at steady-state. Note that the internal potential energy of a LMN with equally probable states at steady-state is zero, whereas its free potential energy equals $-\ln|\mathcal{Y}|$. On the other hand, the internal potential energy of a LMN with "crystalized" behavior around a *unique* ground state is also zero, and the same is true for the free potential energy. Moreover,

$$\overline{\mathcal{P}}_\Omega = \frac{\partial \mathcal{I}_\Omega(\overline{\boldsymbol{y}}^*_\Omega)}{\partial \Omega}, \tag{S49}$$

by virtue of Eqs. (S43) & (S48), whereas

$$\overline{\mathcal{B}}_\Omega = -\Omega \, \frac{\partial^2 \mathcal{I}_\Omega(\overline{\boldsymbol{y}}^*_\Omega)}{\partial \Omega^2}, \tag{S50}$$

by virtue of Eqs. (S44) & (S49). Therefore, the pressure gives the slope of the support curve

$$\sigma_*(\Omega) := \mathcal{I}_\Omega(\overline{\boldsymbol{y}}^*_\Omega), \quad \Omega > 0, \tag{S51}$$

of the self-information of the most likely state at steady-state, whereas the bulk modulus is proportional to the "curvature" of this curve.

In general,

$$\lim_{\Omega\to\infty} \overline{\mathcal{V}}_\Omega = v_\infty\,, \tag{S52}$$

where v_∞ is a constant, given by

$$v_\infty = -\lim_{\Omega\to\infty} \mathrm{E}\left[\ln \frac{\overline{P}_\Omega(\boldsymbol{Y})}{\overline{P}_\Omega(\overline{\boldsymbol{y}}^*_\Omega)}\right], \tag{S53}$$

whereas

$$\lim_{\Omega\to\infty} \overline{\mathcal{P}}^0_\Omega = \lim_{\Omega\to\infty} \overline{\mathcal{P}}_\Omega = \lim_{\Omega\to\infty} \overline{\mathcal{B}}_\Omega = 0\,. \tag{S54}$$

Indeed, Eqs. (S52) & (S53) are a direct consequence of Eqs. (S32) & (S39). On the other hand, Eq. (S52) implies that $\lim_{\Omega\to\infty} \overline{\mathcal{P}}^0_\Omega = 0$ by virtue of Eq. (S42). Note also that

$$\overline{\mathcal{P}}_\Omega = \frac{\partial \ln \overline{Z}_\Omega}{\partial \Omega} = \frac{\partial}{\partial \Omega} \ln\Big[\sum_{\boldsymbol{y}\in\mathcal{Y}} \exp\{-\Omega \overline{V}_\Omega(\boldsymbol{y})\}\Big], \tag{S55}$$

by virtue of Eqs. (S33), (S48) & (S49). This implies

$$\lim_{\Omega\to\infty} \overline{\mathcal{P}}_\Omega = \frac{\partial \ln(1)}{\partial \Omega} = 0, \tag{S56}$$

where we have used the fact that

$$\lim_{\Omega\to\infty} \exp\{-\Omega \overline{V}_\Omega(\boldsymbol{y})\} = \begin{cases} 1, & \text{if } \boldsymbol{y} = \overline{\boldsymbol{y}}^*_\Omega \\ 0, & \text{otherwise}\,. \end{cases} \tag{S57}$$

Clearly, the fact that $\lim_{\Omega\to\infty} \overline{\mathcal{P}}_\Omega = 0$ implies that $\lim_{\Omega\to\infty} \overline{\mathcal{B}}_\Omega = 0$, by virtue of Eq. (S50).

The function

$$V_\infty(\boldsymbol{y};t) := \lim_{\Omega\to\infty} V_\Omega(\boldsymbol{y};t) \tag{S58}$$

is known as large deviation rate function [24]. This function characterizes the decay rate of the probability distribution away from the ground states as $\Omega \to \infty$. Moreover, its steady-state value $\overline{V}_\infty(\boldsymbol{y}) = \lim_{t\to\infty} V_\infty(\boldsymbol{y};t)$ acts as a Lyapunov function for the macroscopic equations (S24) [17,25]. This means that the solution $\boldsymbol{\mu}(t)$ of the macroscopic equations produces a downhill motion in the value of the potential energy landscape $\overline{V}_\infty(\boldsymbol{y})$ until it asymptotically reaches a stable stationary state.

Analytical derivation of $V_\infty(\boldsymbol{y};t)$ is not possible in general. However, when the macroscopic equations are *monostable*, the system size expansion of van Kampen implies that

$$\begin{aligned} V_\infty(\boldsymbol{y};t) &= \lim_{\Omega\to\infty} \frac{1}{2\Omega}\big(\boldsymbol{y}-\boldsymbol{\mu}(t)\big)^T \mathbb{C}^{-1}(t)\big(\boldsymbol{y}-\boldsymbol{\mu}(t)\big) \\ &= \lim_{\Omega\to\infty} \frac{1}{2\Omega}\big(\boldsymbol{y}-\boldsymbol{\mu}(t)\big)^T [\mathbb{N}\mathbb{R}(t)\mathbb{N}]^{-1}\big(\boldsymbol{y}-\boldsymbol{\mu}(t)\big) \\ &= \lim_{N\to\infty} \frac{N_0}{2}\big(\boldsymbol{y}-\boldsymbol{\mu}(t)\big)^T \mathbb{Z}\mathbb{R}^{-1}(t)\mathbb{Z}\big(\boldsymbol{y}-\boldsymbol{\mu}(t)\big) \\ &= \frac{N_0}{2}(\boldsymbol{y}-\boldsymbol{\mu}(t))^T \mathbb{Z}\mathbb{R}^{-1}(t)\mathbb{Z}(\boldsymbol{y}-\boldsymbol{\mu}(t)). \end{aligned} \tag{S59}$$

In these equations, $\boldsymbol{\mu}(t)$ solves the macroscopic equations (S24), the covariance matrix $\mathbb{C}(t)$ equals $\mathbb{N}\mathbb{R}(t)\mathbb{N}$, where $\mathbb{N}$ is a diagonal matrix with elements $1/\sqrt{N_1}, 1/\sqrt{N_2}, \ldots, 1/\sqrt{N_K}$ and $\mathbb{R}(t)$ is the correlation matrix of the random vector $\boldsymbol{W}(t)$ whose elements solve the Lyapunov equations (S30), whereas $\mathbb{Z}$ is a diagonal matrix with elements $\zeta_1, \zeta_2, \ldots, \zeta_K$, where $\zeta_k = N_k/N$. Clearly, $V_\infty(\boldsymbol{y};t)$ is hyper-quadratic in this case around the macroscopic solution $\boldsymbol{\mu}(t)$, which is now the unique ground state. Moreover, the shapes of the equipotential surfaces centered at $\boldsymbol{\mu}(t)$ are ellipsoidal, determined by $\mathbb{R}^{-1}(t)$ and, therefore, by the Lyapunov equations (S30).

If the stationary solution of the master equation can be well-approximated by the LNA method, then

$$\overline{\mathcal{V}}_\Omega \simeq \frac{K}{2}\,, \tag{S60}$$

for those values of Ω at which this is true, where K is the dimension of the state-space $\mathcal{Y}$. Indeed, from Eqs. (S39) & (S59), we have that

$$\begin{aligned}
\overline{\mathcal{V}}_\Omega &\simeq \frac{1}{2}\mathrm{E}\big[(\boldsymbol{Y}-\overline{\boldsymbol{\mu}})^T\overline{\mathbb{C}}^{-1}(\boldsymbol{Y}-\overline{\boldsymbol{\mu}})\big] \\
&= \frac{1}{2}\,\mathrm{tr}\Big[\mathrm{E}\big[(\boldsymbol{Y}-\overline{\boldsymbol{\mu}})^T\overline{\mathbb{C}}^{-1}(\boldsymbol{Y}-\overline{\boldsymbol{\mu}})\big]\Big] \\
&= -\frac{1}{2}\mathrm{E}\Big[\mathrm{tr}\big[(\boldsymbol{Y}-\overline{\boldsymbol{\mu}})^T\overline{\mathbb{C}}^{-1}(\boldsymbol{Y}-\overline{\boldsymbol{\mu}})\big]\Big] \\
&= \frac{1}{2}\mathrm{E}\Big[\mathrm{tr}\big[\overline{\mathbb{C}}^{-1}(\boldsymbol{Y}-\overline{\boldsymbol{\mu}})(\boldsymbol{Y}-\overline{\boldsymbol{\mu}})^T\big]\Big] \\
&= \frac{1}{2}\mathrm{tr}\Big[\overline{\mathbb{C}}^{-1}\mathrm{E}\big[(\boldsymbol{Y}-\overline{\boldsymbol{\mu}})(\boldsymbol{Y}-\overline{\boldsymbol{\mu}})^T\big]\Big] \\
&\simeq \frac{1}{2}\,\mathrm{tr}\big[\overline{\mathbb{C}}^{-1}\overline{\mathbb{C}}\big] = \frac{K}{2}\,,
\end{aligned} \tag{S61}$$

where $\mathrm{tr}[A]$ denotes the trace of a matrix A. To obtain the previous result, we used three well-known properties of the trace: the trace of a scalar is itself, the trace is a linear operator (and therefore it commutes with expectation), and the trace is invariant under cyclic permutations. Moreover, we used the fact that $\mathrm{E}\big[(\boldsymbol{Y}-\overline{\boldsymbol{\mu}})(\boldsymbol{Y}-\overline{\boldsymbol{\mu}})^T\big] \simeq \overline{\mathbb{C}}$ when the LNA method provides a sufficiently accurate solution to the master equation at steady-state.

We can use the pressure as a measure of (thermodynamic) robustness of the LMN with respect to the network size Ω. We say that the LMN is robust against variations in network size if there is no appreciable change in pressure when adding or removing nodes. Therefore, the LMN is robust if the derivative $\partial\overline{\mathcal{P}}_\Omega/\partial\Omega$ of the pressure is small. As a consequence of Eq. (S50), the LMN is robust if the bulk modulus is small (especially at small network sizes). This implies that a robust LMN must significantly resist changes in pressure. On the other hand, Eqs. (S50) & (S51) reveal that the LMN is robust if the network is characterized by a "blunt" self-information curve $\sigma_*(\Omega)$ with small curvature. Note that, if Ω is sufficiently large, then Eq. (S54) implies that the pressure and bulk modulus will approximately be zero and the network will be robust to changes in size. This also implies that the slope of the self-information support curve $\sigma_*(\Omega)$ will approximately be zero and the same will be true for its curvature.

It is important to emphasize here that we can use the bulk modulus $\overline{\mathcal{B}}_\Omega$ to detect network sizes at which the LMN exhibits critical behavior. As a matter of fact, it is well-known that an intensive thermodynamic quantity, such as the pressure, may experience a sharp discontinuity when another thermodynamic variable, such as the network size, varies past a critical value. If the pressure $\overline{\mathcal{P}}_\Omega$ of the LMN experiences such a discontinuity as the network size Ω varies past a critical value Ω_c, then $\overline{\mathcal{B}}_\Omega$ will effectively capture this discontinuity by a delta function located at Ω_c, thus indicating that the network experiences phase transition at Ω_c.

As a consequence of the previous discussion, if the LNA method is valid for large values of Ω, then $\overline{\mathcal{B}}_\Omega \simeq 0$ at these network sizes. Moreover, if the thermodynamic behavior of the LMN changes abruptly when Ω is decreased past a critical value Ω_c, then $\overline{\mathcal{B}}_\Omega$ will produce a sharp spike at this value. A critical network size can demarcate a discontinuous transition of potential wells associated with the ground states. This is a direct consequence of the fact that spike-like behavior in the bulk modulus indicates an abrupt change in the slope of the self-information support curve $\sigma_*(\Omega)$ of the most likely state at steady-state, as predicted by Eq. (S50).

Equilibrium and Non-equilibrium LMNs

We now discuss nuanced technical issues which arise when one is interested in determining the equilibrium status of a LMN.

It can be shown (e.g., see [17,26]) that the entropy of a LMN satisfies the balance equation

$$\frac{d\mathcal{S}_\Omega(t)}{dt} = s_\Omega(t) - h_\Omega(t), \quad t > 0, \tag{S62}$$

where $s_\Omega(t)$ is the *entropy production rate* of the network, defined by

$$s_\Omega(t) := \frac{1}{2} \sum_{k \in \mathcal{K}} \sum_{\boldsymbol{y} \in \mathcal{Y}} \Big[\varphi_k^+(\boldsymbol{y};t) \mathcal{A}_k^+(\boldsymbol{y};t) + \varphi_k^-(\boldsymbol{y};t) \mathcal{A}_k^-(\boldsymbol{y};t) \Big], \tag{S63}$$

whereas $h_\Omega(t)$ is the *heat dissipation rate*, defined by

$$h_\Omega(t) := \frac{1}{2} \sum_{k \in \mathcal{K}} \sum_{\boldsymbol{y} \in \mathcal{Y}} \left\{ \varphi_k^+(\boldsymbol{y};t) \ln\left[\frac{\pi_k^+(\boldsymbol{y} - \widetilde{\boldsymbol{e}}_k)}{\pi_k^-(\boldsymbol{y})} \right] + \varphi_k^-(\boldsymbol{y};t) \ln\left[\frac{\pi_k^-(\boldsymbol{y} + \widetilde{\boldsymbol{e}}_k)}{\pi_k^+(\boldsymbol{y})} \right] \right\}, \tag{S64}$$

with $\widetilde{\boldsymbol{e}}_k$ being the k-th column of the $K \times K$ identity matrix multiplied by N_k^{-1}. In these equations,

$$\varphi_k^+(\boldsymbol{y};t) \; := \; \pi_k^+(\boldsymbol{y} - \widetilde{\boldsymbol{e}}_k) P_\Omega(\boldsymbol{y} - \widetilde{\boldsymbol{e}}_k; t) - \pi_k^-(\boldsymbol{y}) P_\Omega(\boldsymbol{y};t) \tag{S65}$$

$$\varphi_k^-(\boldsymbol{y};t) \; := \; \pi_k^-(\boldsymbol{y} + \widetilde{\boldsymbol{e}}_k) P_\Omega(\boldsymbol{y} + \widetilde{\boldsymbol{e}}_k; t) - \pi_k^+(\boldsymbol{y}) P_\Omega(\boldsymbol{y};t) \tag{S66}$$

are the forward and reverse *probability fluxes* associated with the k-th node of the network, whereas

$$\mathcal{A}_k^+(\boldsymbol{y};t) \; := \; \ln\left[\frac{\pi_k^+(\boldsymbol{y} - \widetilde{\boldsymbol{e}}_k) P_\Omega(\boldsymbol{y} - \widetilde{\boldsymbol{e}}_k; t)}{\pi_k^-(\boldsymbol{y}) P_\Omega(\boldsymbol{y};t)} \right] \tag{S67}$$

$$\mathcal{A}_k^-(\boldsymbol{y};t) \; := \; \ln\left[\frac{\pi_k^-(\boldsymbol{y} + \widetilde{\boldsymbol{e}}_k) P_\Omega(\boldsymbol{y} + \widetilde{\boldsymbol{e}}_k; t)}{\pi_k^+(\boldsymbol{y}) P_\Omega(\boldsymbol{y};t)} \right] \tag{S68}$$

are the *affinities* of the k-th node. The affinities can be viewed as thermodynamic forces that drive the corresponding probability fluxes [17]. The flux $\varphi_k^+(\boldsymbol{y};t)$ quantifies the flow of probability from state $\boldsymbol{y} - \widetilde{\boldsymbol{e}}_k$ to state $\boldsymbol{y}$, whereas the flux $\varphi_k^-(\boldsymbol{y};t)$ quantifies the flow of probability from $\boldsymbol{y} + \widetilde{\boldsymbol{e}}_k$ to $\boldsymbol{y}$. Note that we can write the master equation (S12) as

$$\frac{\partial P_\Omega(\boldsymbol{y};t)}{\partial t} = \sum_{k \in \mathcal{K}} \left\{ \varphi_k^+(\boldsymbol{y};t) + \varphi_k^-(\boldsymbol{y};t) \right\}. \tag{S69}$$

We now consider two notions of equilibrium: dynamic and thermodynamic equilibrium. A LMN is said to reach *dynamic* equilibrium if the joint probability distribution $P_\Omega(\boldsymbol{y};t)$ tends to a stationary distribution $\overline{P}_\Omega(\boldsymbol{y})$ as $t \to \infty$. An irreducible LMN approaches dynamic equilibrium with unique stationary distribution that is strictly positive, asymptotically stable and independent of the initial condition. On the other hand, a LMN is said to reach *thermodynamic* equilibrium if the affinities $\mathcal{A}_k^+(\boldsymbol{y};t)$ and $\mathcal{A}_k^-(\boldsymbol{y};t)$ tend to zero as $t \to \infty$, for every $k \in \mathcal{K}$ and $\boldsymbol{y} \in \mathcal{Y}$. It is clear from Eqs. (S65)–(S68) and Eq. (S69) that thermodynamic equilibrium implies dynamic equilibrium. However, dynamic equilibrium can be reached without the network being in thermodynamic equilibrium. As a matter of fact, a LMN that is in dynamic equilibrium is also in thermodynamic equilibrium only when the following *detailed balance conditions* are satisfied

$$\pi_k^+(\boldsymbol{y} - \widetilde{\boldsymbol{e}}_k) \overline{P}_\Omega(\boldsymbol{y} - \widetilde{\boldsymbol{e}}_k) \; = \; \pi_k^-(\boldsymbol{y}) \overline{P}_\Omega(\boldsymbol{y}) \tag{S70}$$

$$\pi_k^-(\boldsymbol{y} + \widetilde{\boldsymbol{e}}_k) \overline{P}_\Omega(\boldsymbol{y} + \widetilde{\boldsymbol{e}}_k) \; = \; \pi_k^+(\boldsymbol{y}) \overline{P}_\Omega(\boldsymbol{y}), \tag{S71}$$

for every $k \in \mathcal{K}$, $\boldsymbol{y} \in \mathcal{Y}$. One can ensure that a LMN reaches thermodynamic equilibrium by constraining the propensities of a LMN to satisfy a set of well-defined constraints, known as the *Kolmogorov-Wegscheider conditions*; see [17] for details.

It turns out that $0 \leq \overline{s}_\Omega = \overline{h}_\Omega$, where $\overline{s}_\Omega := \lim_{t\to\infty} s_\Omega(t)$ and similarly for $\overline{h}_\Omega$, with equality only when the network is in thermodynamic equilibrium [17, 26]. As a consequence, the rate of entropy production in a LMN at dynamic equilibrium must be equal to the rate of heat dissipation. Moreover, the network is in thermodynamic equilibrium if and only if these rates are zero (i.e., if and only if the network does not produce entropy or dissipates heat).

Living biological systems produce entropy and dissipate heat. As a consequence, these systems must operate away from thermodynamic equilibrium. To check whether this is true for a given LMN model, we can use the maximum absolute affinity at steady-state, given by

$$\bar{\mathcal{A}}_{\max} := \max_{k\in\mathcal{K},\boldsymbol{y}\in\mathcal{Y}} \left\{ |\bar{\mathcal{A}}_k^+(\boldsymbol{y})|, |\bar{\mathcal{A}}_k^-(\boldsymbol{y})| \right\}. \tag{S72}$$

Then, a LMN model is at thermodynamic equilibrium if and only if $\bar{\mathcal{A}}_{\max} = 0$.

Noise-induced Modes, Stochastic Transitions and Bursting

To explain why a noise-induced mode may appear at the origin of the state-space $\mathcal{Y}$ at low population sizes, let $T_e(\boldsymbol{y})$ be the *mean escape time* from a state $\boldsymbol{y} \in \mathcal{Y}$, defined as the average time required for the LMN to move from state $\boldsymbol{y}$ to any other state in $\mathcal{Y}$. Since the fractional activity process is Markovian, governed by the master equation (S12), the time it spends at state $\boldsymbol{y}$ is an exponential random variable with rate parameter $\sum_{k\in\mathcal{K}}[\pi_k^+(\boldsymbol{y}) + \pi_k^-(\boldsymbol{y})]$, which implies that

$$T_e(\boldsymbol{y}) = \frac{1}{\sum_{k\in\mathcal{K}} \left[\pi_k^+(\boldsymbol{y}) + \pi_k^-(\boldsymbol{y})\right]} \; . \tag{S73}$$

Clearly, if $T_e(\boldsymbol{y}) = \infty$, then the state $\boldsymbol{y}$ is *absorbing*. This means that, once the network reaches $\boldsymbol{y}$, it can never leave that state. As a consequence, we can use $T_e(\boldsymbol{y}^*)$ to assess the "stability" of a ground state $\boldsymbol{y}^*$ of the potential energy landscape of the LMN, with higher values of $T_e(\boldsymbol{y}^*)$ indicating that the state $\boldsymbol{y}^*$ is more stable. From Eqs. (S10), (S11) & (S73), we have that

$$\begin{aligned}
[T_e(\boldsymbol{y})]^{-1} &= \sum_{k\in\mathcal{K}} \left[N_k(1-y_k)\lambda_k^+ + N_k(1-y_k)\phi_k(\rho_k(\boldsymbol{y})) + N_k y_k \lambda_k^- + N_k y_k \gamma_k(\rho_k(\boldsymbol{y}))\right] \\
&= N \sum_{k\in\mathcal{K}} \left[\zeta_k(1-y_k)\lambda_k^+ + \zeta_k(1-y_k)\phi_k(\rho_k(\boldsymbol{y})) + \zeta_k y_k \lambda_k^- + \zeta_k y_k \gamma_k(\rho_k(\boldsymbol{y}))\right] \\
&= N \sum_{k\in\mathcal{K}} \zeta_k(1-y_k)\lambda_k^+ + \zeta_k y_k \lambda_k^- \geq 0.
\end{aligned} \tag{S74}$$

As a consequence, when $\lambda_k^+ = 0$ and $\lambda_k^- > 0$, for every $k \in \mathcal{K}$,[1] then $T_e(\boldsymbol{y}) = \infty$ only when $\boldsymbol{y} = \mathbf{0}$. If $T_e(\mathbf{0}) = \infty$, then the master equation (S12) will have a trivial solution $P_\Omega(\boldsymbol{y};t) = \Delta(\boldsymbol{y})$, since the network is initialized at $\mathbf{0}$ and it will never move to another state. In this case, the resulting potential energy landscape $V_\Omega(\boldsymbol{y};t)$ will have a unique global minimum at $\mathbf{0}$ [as a matter of fact, $V_\Omega(\mathbf{0};t) = 0$, whereas $V_\Omega(\boldsymbol{y};t) = \infty$, for every $\boldsymbol{y} \neq \mathbf{0}$].

Since real-world networks must be characterized by non-trivial dynamics, we must have $T_e(\mathbf{0}) < \infty$. The fact that, when $T_e(\mathbf{0}) = \infty$, the probability distribution $P_\Omega(\boldsymbol{y};t)$ is concentrated at the origin suggests that a very large but finite $T_e(\mathbf{0})$ may be indicative of a probability distribution that assigns

[1] This means that a node is leak-free when moving from the inactive to the active state but leaky when moving from the active to the inactive state.

high probability to the zero state, creating a noise-induced mode at the origin of $\mathcal{Y}$. As a matter of fact, we expect that the stationary probability distribution $\overline{P}_\Omega(\boldsymbol{y})$ to have a peak around $\boldsymbol{y} = \mathbf{0}$ whenever the mean escape time of the fractional activity dynamics from the zero state is sufficiently large and the average time it takes for these dynamics to return to the zero state is small (see pages 100 & 101 in [27]).

In general, Eq. (S74) implies that

$$[T_e(\mathbf{0})]^{-1} = N \sum_{k \in \mathcal{K}} \zeta_k \big[\lambda_k^+ + \phi_k(\rho_k(\mathbf{0}))\big], \tag{S75}$$

where $\zeta_k = N_k/N$. Clearly, reducing the net population size N increases $T_e(\mathbf{0})$. It moreover decreases the size of the state-space $\mathcal{Y}$, in which case, there will be fewer states for the network to visit, improving the likelihood of visiting the zero state and thus reducing the average return time to that state. The confluence of these two effects may contribute to the creation of a noise-induced mode at the state of zero fractional activity.

Eq. (S75) shows that, in addition to the net population size N, other system-specific parameters may also influence the mean escape time from the origin. For example, reducing the value of $\lambda_k^+ + \phi_k(\rho_k(\mathbf{0}))$, for every $k \in \mathcal{K}$, will increase the mean time spent at the origin when the LMN reaches complete inactivity.

We should note here that similar arguments can be made to show that, under appropriate conditions, reducing the net population size and tuning system-specific parameters can result in a noise-induced mode at the state $\mathbf{1}$ of maximum fractional activity. Indeed, from Eq. (S74), we have

$$[T_e(\mathbf{1})]^{-1} = N \sum_{k \in \mathcal{K}} \zeta_k \big[\lambda_k^- + \gamma_k(\rho_k(\mathbf{1}))\big]. \tag{S76}$$

This implies that, by reducing N and $\lambda_k^- + \gamma_k(\rho_k(\mathbf{1}))$, for every $k \in \mathcal{K}$, we can increase the mean time spent at $\mathbf{1}$ while improving the likelihood of visiting $\mathbf{1}$ and thus reducing the average return time to this state. As a consequence, the confluence of these two effects may contribute to the creation of a noise-induced mode at the state of maximum fractional activity.

Let us now define a parameter

$$b := \sum_{k \in \mathcal{K}} \zeta_k [\lambda_k^+ + \phi_k(\rho_k(\mathbf{0}))] \geq 0 \tag{S77}$$

and assume that the macroscopic equations (S24) have only one stable fixed point $\boldsymbol{\mu}^*$. Note that, when $b = 0$, the macroscopic equations (S24) imply that the origin of the state-space $\mathcal{Y}$ is also a fixed point (albeit an unstable one) for the macroscopic equations. Thus b is a bifurcation parameter, since the macroscopic equations predict that bifurcation takes place at $b = 0$. Regardless how close the LMN is at the bifurcation point, the macroscopic equations predict that there will be no stable fixed point at the origin, with the dynamics moving away from $\mathbf{0}$ and towards the stable fixed-point $\boldsymbol{\mu}^*$.

On the other hand, our previous discussion implies that, at sufficiently small population sizes, the LMN may behave as if there is a stable fixed point at the origin, in the sense that the network will be operating close to $\mathbf{0}$ with non-negligible probability. This is also true for small nonzero values of the bifurcation parameter b, since Eq. (S75) implies that the mean escape time from the origin is given by $T_e(\mathbf{0}) = (Nb)^{-1}$. This clearly demonstrates that, intrinsic noise present in the network at small population sizes is capable of blurring the bifurcation point from being a single point at $b = 0$ to a small nonnegative neighborhood of 0 while "stabilizing" the unstable fixed point of the macroscopic equations located at the origin of the state-space $\mathcal{Y}$.

Another way to see how intrinsic noise contributes to the creation and stability of a noise-induced mode is by means of the LNA method. For sufficiently large network sizes Ω, the LNA method predicts that the stationary probability distribution $\overline{P}_\Omega(\boldsymbol{y})$ can be sufficiently characterized by a multivariate Gaussian distribution tightly centered around $\boldsymbol{\mu}^*$ with most probability mass being assigned over the state-space $\mathcal{Y}$ which, for all practical purposes, can be thought of as being continuous.[2] As a consequence, the net

[2] Recall that $\mathcal{Y} = \mathcal{Y}_1 \times \mathcal{Y}_2 \times \cdots \times \mathcal{Y}_K$, where $\mathcal{Y}_k := \{0, 1/N_k, \ldots, 1\}$.

probability mass assigned by this distribution over the state-space $\mathcal{Y}$ will approximately equal to 1, as expected. However, as the network size Ω decreases, the Gaussian distribution becomes wider around $\boldsymbol{\mu}^*$ and may appreciably extend beyond the state-space $\mathcal{Y}$, which will be discrete for small enough Ω. In this case, the net probability mass assigned at values outside the state-space $\mathcal{Y}$ will not be negligible, and the net probability mass assigned over $\mathcal{Y}$ will be smaller than one. As we explained above, and under appropriate conditions, the Gaussian approximation will begin to break down by placing significant probability mass outside of $\mathcal{Y}$. This may force the stationary distribution $\overline{P}_\Omega(\boldsymbol{y})$ to undergo a qualitative change where the lost probability mass may be thought of as being absorbed at the origin, creating a mode in $\overline{P}_\Omega(\boldsymbol{y})$ at $\mathbf{0}$.

From a potential energy landscape perspective, the width of the potential well associated with the peak of the stationary probability distribution at $\boldsymbol{\mu}^*$ will increase as Ω decreases, whereas its depth will decrease. This behavior is also influenced by other system-specific parameters that control the steady-state solution $\overline{\mathbb{C}}$ of the Lyapunov equations (S30). On the other hand, the width and depth of the potential well associated with the peak of the stationary probability distribution at $\mathbf{0}$ will both increase as Ω decreases. If the network parameters are such that the potential well at $\boldsymbol{\mu}^*$ is sufficiently wide and shallow, then a state within this potential well will eventually move towards the potential well at $\mathbf{0}$ with high probability and stay there for an appreciable amount of time before exiting.

The previous discussion provides a clear explanation of the fact that intrinsic noise is an important factor for bursting. In addition to Ω (or N) and $\overline{\mathbb{C}}$, this behavior also depends on how far $\boldsymbol{\mu}^*$ is from the origin $\mathbf{0}$, since bursting is clearly better pronounced when $\boldsymbol{\mu}^*$ is further away from the origin. In general however this requires a wider potential well at $\boldsymbol{\mu}^*$. Hence, the network size Ω and any other system-specific parameter that affects the steady-state solution $\boldsymbol{\mu}^*$ of the macroscopic equations (S24) and the steady-state solution $\overline{\mathbb{C}}$ of the Lyapunov equations (S30) will also affect bursting. This allows the LMN to control bursting by employing alternative strategies.

Defining Avalanches

A common way to define avalanches has originated from work on neural networks, since the emergence of avalanches is a fundamental property of such networks [28,29]. This definition is based on partitioning time into bins $\mathcal{T}_i = [i\Delta t, (i+1)\Delta t)$, $i = 0, 1, \ldots$, of equal duration $\Delta t > 0$ and associating to each bin $\mathcal{T}_i$ a frame $\mathcal{F}_i$, defined as the portion $\{\boldsymbol{X}(t), t \in \mathcal{T}_i\}$ of the activity process during $\mathcal{T}_i$. The frame $\mathcal{F}_i$ is said to be *blank* if the activity process $\boldsymbol{X}(t)$ is zero within $\mathcal{T}_i$; otherwise, the frame $\mathcal{F}_i$ is said to be *active*. Then, an avalanche is defined to be a sequence of consecutively active frames that is preceded and ended by a blank frame [28]. Note that, if

$$\mathcal{H}(t) := \frac{1}{N} \sum_{n \in \mathcal{N}} X_n(t) = \sum_{k \in \mathcal{K}} \zeta_k Y_k(t), \tag{S78}$$

where $\zeta_k = N_k/N$, then $\mathcal{F}_i$ is blank if $\max_{t \in \mathcal{T}_i}\{\mathcal{H}(t)\} \leq \epsilon$, whereas $\mathcal{F}_i$ is active if $\max_{t \in \mathcal{T}_i}\{\mathcal{H}(t)\} > \epsilon$. Note that $\mathcal{H}(t)$ is the net fractional activity of the activity process $\boldsymbol{X}(t)$ at time t. As a consequence, $\epsilon \geq 0$ is a small threshold that dictates the minimum percentage of nodes that can be active in the network in order for the network to be deemed active.

It is common to characterize an avalanche by two parameters: its duration and size. The *duration* D of an avalanche is defined to be the number of consecutively active frames multiplied by Δt. On the other hand, its *size* Σ is simply the number of times within the duration D that an element of the activity process $\boldsymbol{X}(t)$ becomes active (switches from 0 to 1).

Unfortunately, the previous definitions depend on the choice of Δt. Moreover, the definitions are sensitive to arbitrary shifts of the time axis. For this reason, we provide here an alternative definition for an avalanche that is not influenced by the previous factors. In particular, we say that an avalanche occurs within a time window $[t, t+\tau)$, whenever the following three conditions are satisfied:

(i) There exist some small $dt > 0$ such that $\mathcal{H}(t') \leq \epsilon$, for all $t' \in [t - dt, t)$; i.e., the network is inactive immediately before time t.

(ii) $\mathcal{H}(t') > \epsilon$, for all $t' \in [t, t+\tau)$; i.e., the network is active during the time interval $[t, t+\tau)$.

(iii) $\mathcal{H}(t+\tau) \leq \epsilon$; i.e., the network becomes inactive at time $t+\tau$.

Using the previous definition, it is not difficult to see that the duration D is now given by τ, whereas the size is defined as before by replacing D by τ. Note that a given element of the activity process may transition from 0 to 1 multiple times throughout the duration. As a consequence, the size of an avalanche is not limited by N. To account for the effect of population size, it is common to consider the fractional avalanche size Σ/N [29].

Epidemiological Example

In epidemiology, a common model of disease spreading is the SIS model [30]. According to this model, the n-th individual in a directed weighted network $\mathcal{G}$ of N interacting individuals is assumed to be in one of two states with respect to a disease at time t: *susceptible* (S) and *infected* (I). In this case, the state of the epidemiological system at time t is characterized by a random vector $\boldsymbol{X}(t)$ whose n-th element $X_n(t)$ takes value 1, if the n-th individual is infected, and 0, if she is susceptible. It is assumed that recovery from infection does not confer resistance to the disease with infected individuals becoming susceptible after recovery. For example, bacterial infection is modeled well by the SIS model, since an individual who recovers (e.g., through the use of antibiotics) is susceptible to re-infection.

In the classical SIS model, infection can only be transmitted from an infected to a susceptible individual. Note however that some infections can be acquired from other sources, such as the environment, animals, terror attacks, or self-infection. For example, individuals may be colonized by bacteria and be healthy for long periods of time until the bacteria suddenly seize the opportunity to pathogenically infect the individual. As a consequence, we choose here to discuss a slightly more general version of the SIS model known as the SISa model [31,32].

Although the SISa model is general enough to describe a variety of infections (e.g., due to illnesses, computer viruses, or social contagions), we focused on a matter of pressing concern to public health: infections due to methicillin resistant *staphylococcus aureus* (MRSA). We use the SISa model as a simple model of MRSA outbreaks in which bacterial infections, due to self-infection, are possible and the number of individuals spreading MRSA infection can be small, in which case stochastic modeling is necessary [33]. MRSA has been studied in confined swine populations, where more invasive and comprehensive data collection is feasible [34].

We assume that the propensity by which the n-th individual transitions from the susceptible to the infected state depends on a net input $r_n(\boldsymbol{x}) = h_n + \boldsymbol{a}_n^T \boldsymbol{x}$, where $h_n \geq 0$ is the propensity of the individual to become infected regardless of her social contacts and $\boldsymbol{a}_n^T$ is the n-th row of the adjacency matrix $\mathbb{A}$ of the underlying network of infectious social contacts. The element $a_{nn'}$ of the adjacency matrix provides the rate at which the n-th susceptible individual will be infected by the n'-th infected individual and, as such, it is assumed to be nonnegative; i.e., $a_{nn'} \geq 0$, for every $n, n' \in \mathcal{N}$, with $a_{nn} = 0$, for every $n \in \mathcal{N}$. Clearly, $r_n(\boldsymbol{x})$ represents the total infectious influence to the n-th individual. As a consequence, we set $p_n^+(\boldsymbol{x}) = (1 - x_n) r_n(\boldsymbol{x})$. On the other hand, we assume that the propensity of the n-th infected individual to recover is constant, given by ℓ_n^-, which implies that $p_n^-(\boldsymbol{x}) = \ell_n^- x_n$.

To simplify the previous model, we assume one homogeneous population of individuals and study the fraction $Y(t)$ of the population that is infected at time t, given by

$$Y(t) = \frac{1}{N} \sum_{n \in \mathcal{N}} X_n(t). \tag{S79}$$

In this case, one finds that $Na_{nn'} = w > 0$, for every $n, n' \in \mathcal{N}$ such that $n \neq n'$, whereas $h_n = \eta > 0$, $\ell_n^+ = 0$, and $\ell_n^- = \lambda > 0$, for every $n \in \mathcal{N}$. This implies an all-to-all connectivity, which can be justified by considering the fact that, in small populations, it is always possible for any two individuals to come in contact with each other. We take $w > 0$, because sick individuals can only spread infection, whereas we take $\eta > 0$, because infections may be acquired independently of the network state.

The fractional activity process $\{Y(t), t \geq 0\}$ is Markovian, governed by the following master equation:

$$\frac{\partial P(y;t)}{\partial t} = \pi^+(y - 1/N)P(y - 1/N;t) + \pi^-(y + 1/N)P(y + 1/N;t) - \big[\pi^+(y) + \pi^-(y)\big]P(y;t), \quad \text{(S80)}$$

initialized with $P(y;0) = \Delta(y)$, where $P(y;t) := \Pr[Y(t) = y \mid Y(0) = 0]$ and

$$\pi^+(y) = N(1 - y)(\eta + wy) \quad \text{(S81)}$$

$$\pi^-(y) = N\lambda y. \quad \text{(S82)}$$

Moreover, the macroscopic equation (S24) is given by

$$\frac{d\mu(t)}{dt} = \big[1 - \mu(t)\big]\big[\eta + w\mu(t)\big] - \lambda\mu(t), \quad \text{(S83)}$$

with initial condition $\mu(0) = 0$, whereas the Lyapunov equation (S30) for the noise variance in the LNA method is given by

$$\frac{dr(t)}{dt} = \big[1 - \mu(t)\big]\big[\eta + w\mu(t)\big] + \lambda\mu(t) + 2\big\{w\big[1 - \mu(t)\big] - \big[\eta + w\mu(t)\big] - \lambda\big\}r(t), \quad \text{(S84)}$$

initialized by $r(0) = 0$. This equation is driven by $\mu(t)$ that solves Eq. (S83). Note that Eq. (S83) has a unique fixed point μ^*, such that $0 < \mu^* < 1$, given by (recall that $w, \eta > 0$)

$$\mu^* = [(w - \lambda - \eta) + \sqrt{(w - \lambda - \eta)^2 + 4w\eta})]/2w,$$

which can be shown to be stable.

Using data from a Danish swine herd, the parameters w and λ have been estimated to take values $w = 0.108$ days^{-1} and $\lambda = 0.0571$ days^{-1} [34]. However, the parameter η could not be reliably estimated from these data. To illustrate the case when infections are rarely contracted (i.e., the case when the environment is relatively clean but not completely free of MRSA), we set $\eta = 10^{-4}$ days^{-1}. From Eq. (S81), note that $\pi^+(y) > 0$, when $\eta > 0$, $y < 1$. Therefore, $0 \to 1$ and Proposition 2 implies that $Y(t)$ is irreducible.

Because the system is one-dimensional, its state space is reasonably sized. It was therefore possible to numerically solve the master equation (S80) for $P_\Omega(y;t)$ using the Krylov subspace approximation (KSA) method implemented by the Expokit software package [35]. To do so, we used a tight tolerance parameter of 10^{-6} and a value $K_0 = 30$ for the dimension of the Krylov subspace. We then employed Eq. (S32) to evaluate the potential energy landscape $V_\Omega(y;t)$ and used the solution to the master equation after 30 years as an approximation to the stationary probability distribution $\overline{P}_\Omega(y)$. From this distribution, we numerically evaluated the internal potential energy $\overline{\mathcal{V}}_\Omega$ and entropy $\overline{\mathcal{S}}_\Omega$ at steady-state. We then calculated the stationary free potential energy according to $\overline{\mathcal{A}}_\Omega = \overline{\mathcal{V}}_\Omega - \overline{\mathcal{S}}_\Omega$. We set $N_0 = 200$, in which case, $\Omega = N/200$. By evaluating $\overline{\mathcal{A}}_\Omega$ for $\Omega = 0.005, 0.01, \ldots, 1$, we computed the stationary pressure $\overline{\mathcal{P}}_\Omega$ using Eq. (S43) and subsequently the bulk modulus $\overline{\mathcal{B}}_\Omega$ using Eq. (S44). We approximated all derivatives with respect to Ω using backward differences with $\Delta\Omega = 0.005$. When required, we drew sample trajectories from the master equation using the exact Gillespie algorithm [36, 37]. Finally, we numerically solved the macroscopic equation (S83) and the Lyapunov equation (S84) using the stiff 'ode23s' solver in MATLAB® with the default parameters. This resulted in a unique and stable macroscopic stationary state $\mu^* = 0.4719$.

One may be curious about the rather long time scales involved in Fig. 4 of the Main Text. The infection dynamics occur on the order of months (e.g., an infected individual requires an average time of $1/\lambda = 17.5$ days to recover). However, the long-term infection trends (i.e., outbreaks of infections that eventually die out, only for another outbreak to occur) take place on the time span of multiple years. Recall that the system is initialized with all individuals susceptible and none infected, and therefore nothing happens in the model until an infection is acquired from the environment. This process is captured by the parameter η which is very small, indicating that we are modeling a system where the environment (e.g., the pig pen) is kept relatively clean. Quantitatively, one sees from Eq. (S81) that $\pi^+(0) = N\eta$, and thus the start of a new outbreak is a rare-event for small N and η. Therefore, over a time frame of many years, one may observe multiple outbreaks of MRSA infections.

Neural Network Example

A model that fits well within our framework has been put forth in the literature to explain biological neural networks [16]. This model is based on an interconnected directed weighted network $\mathcal{G}$ of N neurons in a set $\mathcal{N} = \{1, 2, \ldots, N\}$ that can exist in one of two distinct states: an active state, during which a neuron fires an action potential,[3] and a quiescent state, during which a neuron is at rest. In this case, the state of the neural system at time t is characterized by a random vector $\boldsymbol{X}(t)$ whose n-th element $X_n(t)$ takes value 1, if the n-th neuron is active at time t, and 0 otherwise.

Here, we study the stochastic behavior of a group of interacting neurons embedded within a larger neural network [38]. We assume that, when the n-the neuron is inactive, it is driven to become active by a net input $r_n(\boldsymbol{x}) = h_n + \boldsymbol{a}_n^T \boldsymbol{x}$, where $h_n > 0$ is the external input to the neuron that quantifies the influence of surrounding neurons and external environmental factors, and $\boldsymbol{a}_n^T$ is the n-th row of the adjacency matrix $\mathbb{A}$ of the network. If no external input is present, h_n may be chosen to account for the (small) rate at which a neuron might fire independently of the incoming synaptic input. The element $a_{nn'}$ of the adjacency matrix $\mathbb{A}$ provides the synaptic weight from the n'-th to the n-th neuron. If $a_{nn'} > 0$, then the n'-th neuron *excites* the n-th neuron making it more likely to spike, whereas, if $a_{nn'} < 0$, then the n'-th neuron *inhibits* the n-th neuron making it less likely to spike. Finally, $a_{nn'} = 0$ indicates that the n-th neuron has no synaptic input from the n'-th neuron. It is assumed that a neuron does not regulate itself, in which case $a_{nn} = 0$, for $n \in \mathcal{N}$.

The propensity by which the n-th neuron transitions from the quiescent to the active state is assumed to monotonically depend on the total synaptic input $r_n(\boldsymbol{x})$ to the neuron by means of a function $f_n(r) = \langle r > 0 \rangle \tanh(r)$, where $\langle r > 0 \rangle$ is the Iverson bracket, taking value 1, if $r > 0$, and 0 otherwise. In this case, $p_n^+(\boldsymbol{x}) = (1 - x_n)\langle r_n(\boldsymbol{x}) > 0 \rangle \tanh(r_n(\boldsymbol{x}))$, where we take $\ell_n^+ = 0$. On the other hand, the propensity of the n-th neuron to transition from the active to the quiescent state is assumed to be a constant ℓ_n^-, regardless of the system state, which implies that $p_n^-(\boldsymbol{x}) = \ell_n^- x_n$.

We simplify the previous model by assuming that the neural network under consideration consists of two homogeneous populations $\mathcal{N}_1$ and $\mathcal{N}_2$ of excitatory and inhibitory neurons, respectively. The fractional activity process $\boldsymbol{Y}(t)$ is now two-dimensional, with elements $Y_1(t)$ and $Y_2(t)$ given by

$$Y_1(t) = \frac{1}{N_1} \sum_{n \in \mathcal{N}_1} X_n(t), \quad Y_2(t) = \frac{1}{N_2} \sum_{n \in \mathcal{N}_2} X_n(t). \tag{S85}$$

Due to homogeneity, we find that $N_1 a_{nn'} = w_{11} > 0$, for every $n, n' \in \mathcal{N}_1$ such that $n \neq n'$, $N_2 a_{nn'} = w_{22} < 0$, for every $n, n' \in \mathcal{N}_2$ such that $n \neq n'$, $N_1 a_{nn'} = w_{21} > 0$, for every $n \in \mathcal{N}_2$, $n' \in \mathcal{N}_1$, and $N_2 a_{nn'} = w_{12} < 0$, for every $n \in \mathcal{N}_1$, $n' \in \mathcal{N}_2$. Moreover, $h_n = \eta_1 > 0$, for every $n \in \mathcal{N}_1$, $h_n = \eta_2 > 0$, for every $n \in \mathcal{N}_2$, $\ell_n^+ = 0$, $\ell_n^- = \lambda_1 > 0$, for every $n \in \mathcal{N}_1$, and $\ell_n^+ = 0$, $\ell_n^- = \lambda_2 > 0$, for every $n \in \mathcal{N}_2$. Note that the implied all-to-all connectivity is a common assumption in the neuroscience literature [16, 39].

[3] The active state includes the accompanying refractory period wherein the neuron is hyperpolarized.

It is usually justified by noting that some regions of the brain are comprised of neurons that are highly interconnected among themselves.

To simplify matters further, we set $N_1 = N_2 = N/2$, $w_{11} = w_{21} = w_e > 0$, $w_{12} = w_{22} = w_i < 0$, $\lambda_1 = \lambda_2 = \lambda > 0$, $\eta_1 = \eta_2 = \eta > 0$, and $\phi_1(\rho) = \phi_2(\rho) = \phi(\rho) = \langle \rho > 0 \rangle \tanh(\rho)$. This implies that $\rho_1(\boldsymbol{y}) = \rho_2(\boldsymbol{y}) = \rho(\boldsymbol{y}) = w_e y_1 + w_i y_2 + \eta$. In this case, the fractional activity process $\{\boldsymbol{Y}(t), t \geq 0\}$ is Markovian, governed by the master equation (S12) with propensity functions

$$\pi_1^+(y_1, y_2) = \frac{N}{2}(1 - y_1)\langle \eta + w_e y_1 + w_i y_2 > 0 \rangle \tanh(\eta + w_e y_1 + w_i y_2) \tag{S86}$$

$$\pi_2^+(y_1, y_2) = \frac{N}{2}(1 - y_2)\langle \eta + w_e y_1 + w_i y_2 > 0 \rangle \tanh(\eta + w_e y_1 + w_i y_2) \tag{S87}$$

$$\pi_1^-(y_1, y_2) = \frac{N}{2}\lambda y_1 \tag{S88}$$

$$\pi_2^-(y_1, y_2) = \frac{N}{2}\lambda y_2. \tag{S89}$$

Moreover, the macroscopic equations (S24) are found to be

$$\frac{d\mu_1(t)}{dt} = \big[1 - \mu_1(t)\big]\langle w_e\mu_1(t) + w_i\mu_2(t) + \eta > 0 \rangle \tanh(w_e\mu_1(t) + w_i\mu_2(t) + \eta) - \lambda\mu_1(t) \tag{S90}$$

$$\frac{d\mu_2(t)}{dt} = \big[1 - \mu_2(t)\big]\langle w_e\mu_1(t) + w_i\mu_2(t) + \eta > 0 \rangle \tanh(w_e\mu_1(t) + w_i\mu_2(t) + \eta) - \lambda\mu_2(t), \tag{S91}$$

initialized by $\mu_1(0) = \mu_2(0) = 0$. Finally, the Lyapunov equations (S30) for the noise correlations in the LNA method can be determined by specifying the diffusion terms as

$$D_1(\mu_1, \mu_2) = (1 - \mu_1)\langle w_e\mu_1 + w_i\mu_2 + \eta > 0 \rangle \tanh(w_e\mu_1 + w_i\mu_2 + \eta) + \lambda\mu_1 \tag{S92}$$

$$D_2(\mu_1, \mu_2) = (1 - \mu_2)\langle w_e\mu_1 + w_i\mu_2 + \eta > 0 \rangle \tanh(w_e\mu_1 + w_i\mu_2 + \eta) + \lambda\mu_2, \tag{S93}$$

and the derivatives of the drift terms as

$$\begin{aligned} A_{11}(\mu_1, \mu_2) &= -\lambda + \langle w_e\mu_1 + w_i\mu_2 + \eta > 0 \rangle \\ &\quad \times \big\{ w_e(1 - \mu_1)\big[1 - \tanh^2(w_e\mu_1 + w_i\mu_2 + \eta)\big] - \tanh(w_e\mu_1 + w_i\mu_2 + \eta) \big\} \end{aligned} \tag{S94}$$

$$A_{12}(\mu_1, \mu_2) = \langle w_e\mu_1 + w_i\mu_2 + \eta > 0 \rangle \, w_i(1 - \mu_1)\big[1 - \tanh^2(w_e\mu_1 + w_i\mu_2 + \eta)\big] \tag{S95}$$

$$A_{21}(\mu_1, \mu_2) = \langle w_e\mu_1 + w_i\mu_2 + \eta > 0 \rangle \, w_e(1 - \mu_2)\big[1 - \tanh^2(w_e\mu_1 + w_i\mu_2 + \eta)\big] \tag{S96}$$

$$\begin{aligned} A_{22}(\mu_1, \mu_2) &= -\lambda + \langle w_e\mu_1 + w_i\mu_2 + \eta > 0 \rangle \\ &\quad \times \big\{ w_i(1 - \mu_2)\big[1 - \tanh^2(w_e\mu_1 + w_i\mu_2 + \eta)\big] - \tanh(w_e\mu_1 + w_i\mu_2 + \eta) \big\}. \end{aligned} \tag{S97}$$

By considering the fact that $\eta > 0$ and $w_s + \eta > 0$, and by employing the change of variables:

$$\begin{pmatrix} \mu_1' \\ \mu_2' \end{pmatrix} = \begin{pmatrix} 1/2 & 1/2 \\ 1/2 & -1/2 \end{pmatrix} \begin{pmatrix} \mu_1 \\ \mu_2 \end{pmatrix} \quad \Leftrightarrow \quad \begin{pmatrix} \mu_1 \\ \mu_2 \end{pmatrix} = \begin{pmatrix} 1 & 1 \\ 1 & -1 \end{pmatrix} \begin{pmatrix} \mu_1' \\ \mu_2' \end{pmatrix},$$

we can show that Eqs. (S90) & (S91) have a unique fixed point $(\mu_1^*, \mu_2^*) = (\mu^*, \mu^*)$, where μ^* is the unique solution of the nonlinear equation

$$\langle w_s\mu + \eta > 0 \rangle \tanh(w_s\mu + \eta) = \frac{\lambda\mu}{1 - \mu}$$

that satisfies $0 < \mu^* < 1$. It turns out that the resulting fixed point is stable [16].

When $\eta + w_e + w_i > 0$, we have that $\mathbf{0} \to \mathbf{1}$ by following a sequence of state transitions whereby all excitatory neurons become active one-by-one and all inhibitory neurons become active one-by-one. As a consequence, $\boldsymbol{Y}(t)$ will be irreducible in this case according to Proposition 2.

By following [16], we set $\lambda = 0.1\,\mathrm{ms}^{-1}$ and $\eta = 0.001$. We also defined two new parameters w_s and w_d, given by

$$w_s := w_e + w_i \quad \text{and} \quad w_d := w_e - w_i. \tag{S98}$$

Note that $w_s < w_d$, since $w_i < 0$. Moreover, $\boldsymbol{Y}(t)$ is irreducible for $w_s + \eta > 0$. It turns out that the steady-state solution of the macroscopic equations (S90) & (S91) depends only on w_s, whereas bursting is controlled by the value of w_d. By following [16], we set $w_s = 0.2$ and study the behavior of the neural network for various values of w_d. We start with $w_d = 0.3$, in which case the network is not balanced (i.e., $w_s \not\ll w_d$), and proceed to examine the effect of w_d.

To do so, we numerically solved the corresponding master equation for the joint probability distribution $P_\Omega(y_1, y_2; t)$ using the KSA method with tolerance parameter of 10^{-30} and a value $K_0 = 50$ for the dimension of the Krylov subspace. We took the value of the tolerance parameter to be appreciably smaller than in the case of the SISa model in order to effectively deal with the increased dimensionality of the state-space $\mathcal{Y}$. Moreover, we took the value of K_0 to be larger than the one used in the case of the SISa model in order to effectively deal with the increased cardinality of $\mathcal{Y}$. Similarly to the case of the SISa model, we employed Eq. (S32) to evaluate the stationary potential energy landscape $\overline{V}_\Omega(y_1, y_2)$. We set $N_0 = 200$, in which case, $\Omega = N/200$, and used the solution to the master equation at 2,000 ms as an approximation to the stationary probability distribution $\overline{P}_\Omega(y_1, y_2)$, since we noticed that the neural network is approximately at steady-state after that time. By using this distribution, we numerically evaluated the internal potential energy $\overline{\mathcal{V}}_\Omega$ and entropy $\overline{\mathcal{S}}_\Omega$ at steady-state. We then calculated the stationary free potential energy according to $\overline{\mathcal{A}}_\Omega = \overline{\mathcal{V}}_\Omega - \overline{\mathcal{S}}_\Omega$. By evaluating $\overline{\mathcal{A}}_\Omega$ for $\Omega = 0.01, 0.02, \ldots, 1$, we computed the stationary pressure $\overline{\mathcal{P}}_\Omega$ using Eq. (S43) and subsequently the bulk modulus $\overline{\mathcal{B}}_\Omega$ using Eqs. (S44). We approximated all derivatives with respect of Ω using backward differences with $\Delta\Omega = 0.01$.[4] When required, we drew sample trajectories from the master equation using the exact Gillespie algorithm. Finally, we numerically solved the macroscopic equations (S90) & (S91) and the corresponding Lyapunov equations using the stiff 'ode23s' solver in MATLAB® with the default parameters, which resulted in a unique and stable macroscopic stationary state $\boldsymbol{\mu}^* = (\mu_1^*, \mu_2^*)^T = (0.5032, 0.5032)^T$.

References

[1] Kauffman SA (1969) Metabolic stability and epigenesis in randomly constructed genetic nets. Journal of Theoretical Biology 22: 437–467.

[2] Kauffman SA (1993) The origins of order. New York, NY: Oxford University Press.

[3] Shmulevich I, Dougherty ER, Kim S, Zhang W (2002) Probabilistic Boolean networks: a rule-based uncertainty model for gene regulatory networks. Bioinformatics 18(2): 261–274.

[4] Shmulevich I, Dougherty ER, Zhang W (2002) From Boolean to probabilistic Boolean networks as models of genetic regulatory networks. Proceedings of the IEEE 90(11): 1778–1792.

[5] Greil F, Drossel B (2005) Dynamics of critical Kauffman networks under asynchronous stochastic update. Physical Review Letters 95: 048701.

[4]Since we are interested in the behavior of a LMN *en route* to the thermodynamic limit, we must take $\Delta\Omega$ to be the minimum allowable change in network size such that $\Delta N \zeta_k = N_0 \Delta\Omega \zeta_k$ is integer valued, for all $k = 1, 2, \ldots, K$, where $\zeta_k := N_k/N$ is fixed. Since $K = 2$ and $N_1 = N_2 = N/2$, we have $\zeta_1 = \zeta_2 = 1/2$ and, therefore, we set $\Delta\Omega = 0.01$.

[6] Shreim A, Berdahl A, Greil F, Davidson J, Paczuski M (2010) Attractor and basin entropies of random boolean networks under asynchronous stochastic update. Physical Review E 82: 035102(R).

[7] Yang M, Chu T (2012) Evaluation of attractors and basins of asynchronous random Boolean networks. Physical Review E 85: 056105.

[8] Siebert H, Bockmayr A (2008) Temporal constraints in the logical analysis of regulatory networks. Theoretical Computer Science 391(3): 258–275.

[9] Abou-Jaoudé W, Ouattara DA, Kaufman M (2009) From structure to dynamics: Frequency tuning in the p53-Mdm2 network. I. Logical approach. Journal of Theoretical Biology 258(4): 561–577.

[10] Vahedi G, Faryabi B, Chamberland J, Datta A, Dougherty ER (2009) Sampling rate-dependent probabilistic Boolean networks. Journal of Theoretical Biology 261(4): 540–547.

[11] Bauer AL, Jackson TL, Jiang Y, Rohlf T (2010) Receptor cross-talk in angiogenesis: Mapping environmental cues to cell phenotype using a stochastic, Boolean signaling network model. Journal of Theoretical Biology 264(3): 838–846.

[12] Sevim V, Gong X, Socolar JES (2010) Reliability of transcriptional cycles and the yeast cell-cycle oscillator. PLoS Computational Biology 6(7): e1000842.

[13] Teraguchi S, Kumagai Y, Vandenbon A, Akira S, Standley DM (2011) Stochastic binary modeling of cells in continuous time as an alternative to biochemical reaction equations. Physical Review E 84: 062903.

[14] Stoll G, Viara E, Barillot E, Calzone L (2012) Continuous time Boolean modeling for biological signaling: application of Gillespie algorithm. BMC Systems Biology 6: 116.

[15] van Kampen NG (2007) Stochastic processes in physics and chemistry. Amsterdam: Elsevier, 3rd edition.

[16] Benayoun M, Cowan JD, van Drongelen W, Wallace E (2010) Avalanches in a stochastic model of spiking neurons. PLoS Computational Biology 6(7):e1000846.

[17] Goutsias J, Jenkinson G (2013) Markovian dynamics on complex reaction networks. Physics Reports 529(2): 199–264.

[18] Alberty RA (2003) Thermodynamics of biochemical reactions. Hoboken, NJ: Wiley-Interscience.

[19] Dill KA, Bromberg S (2011) Molecular driving forces: Statistical thermodynamics in biology, chemistry, physics, and nanoscience. New York, NY: Galand Science, 2nd edition.

[20] Haddad WM, Chellaboina V, Nersesov SG (2005) Thermodynamics: A dynamical systems approach. Princeton, NJ: Princeton University Press.

[21] Ross J (2008) Thermodynamics and fluctuations far from equilibrium. Berlin: Springer-Verlag.

[22] Cover TM, Thomas JA (1991) Elements of information theory. New York, NY: John Wiley & Sons.

[23] Qian H (2009) Entropy demystified: The "thermo"-dynamics of stochastically fluctuating systems. In: Michael LJ, Brand L, editors, Methods in Enzymology, San Diego, CA: Elsevier, volume 467. pp. 111–134.

[24] Touchette H (2009) The large deviation approach to statistical mechanics. Physics Reports 478(1-3): 1–69.

[25] Zhou D, Qian H (2011) Fixation, transient landscape, and diffusion dilemma in stochastic evolutionary game dynamics. Physical Review E 84: 031907.

[26] Ge H, Qian H (2010) Physical origins of entropy production, free energy dissipation, and their mathematical representations. Physical Review E 81: 051133.

[27] Stroock DW (2005) An introduction to Markov processes. Berlin: Springer.

[28] Beggs JM, Plenz D (2003) Neuronal avalanches in neocortical circuits. Journal of Neuroscience 23: 11167–11177.

[29] Klaus A, Yu S, Plenz D (2011) Statistical analyses support power law distributions found in neuronal avalanches. PLoS One 6: e19779.

[30] Newman MEJ (2010) Networks: An introduction. New York, NY: Oxford University Press.

[31] Hill AL, Rand DG, Nowak MA, Christakis NA (2010) Emotions as infectious diseases in a large social network: the SISa model. Proceedings of the Royal Society B 277: 3827–3835.

[32] Hill AL, Rand DG, Nowak MA, Christakis NA (2010) Infectious disease modeling of social contagion in networks. PLoS Computational Biology 6(11): e1000968.

[33] Austin DJ, Anderson RM (1999) Studies of antibiotic resistance within the patient, hospitals and the community using simple mathematical models. Philosophical Transactions of the Royal Society of London B 354: 721–738.

[34] Broens EM, Espinosa-Gongora C, Graat EAM, Vendrig N, Van Der Wolf PJ, et al. (2012) Longitudinal study on transmission of MRSA CC398 within pig herds. BMC Veterinary Research 8: 58.

[35] Sidje RB (1998) EXPOKIT: A software package for computing matrix exponentials. ACM Transactions on Mathematical Software 24(1): 130–156.

[36] Gillespie DT (1977) Exact stochastic simulation of coupled chemical reactions. Journal of Physical Chemistry 81(25): 2340–2361.

[37] Gillespie DT (1992) A rigorous derivation of the chemical master equation. Physica A 188: 404–425.

[38] Koch C, Segev I (1998) Methods in neural modeling: From ions to networks. Cambridge, MA: MIT Press, 2nd edition.

[39] Bressloff PC (2010) Metastable states and quasicycles in a stochastic Wilson-Cowan model of neural population dynamics. Physical Review E 82: 051903.
